# The Mechano-Ubiquitinome of Articular Cartilage: Differential Ubiquitination and Activation of a Group of ER-Associated DUBs and ER Stress Regulators

**DOI:** 10.1016/j.mcpro.2022.100419

**Published:** 2022-09-28

**Authors:** Nitchakarn Kaokhum, Adán Pinto-Fernández, Mark Wilkinson, Benedikt M. Kessler, Heba M. Ismail

**Affiliations:** 1 Department of Infection, Immunity and Cardiovascular Disease, University of Sheffield, Sheffield, UK; 2 Nuffield Department of Medicine, Target Discovery Institute, Centre for Medicines Discovery, University of Oxford, Oxford, UK; 3 Department of Oncology and Metabolism, University of Sheffield, Sheffield, UK; 4 Healthy Lifespan Institute (HELSI), University of Sheffield, Sheffield, UK

**Keywords:** mechanical injury, ubiquitin remnant, articular cartilage, ubiquitomics, ER stress, ERAD response, DUB, deubiquitinase, ER, endoplasmic reticulum, ERAD, ER-associated degradation, MCP, metacarpophalangeal, MS/MS, tandem MS, PBST, 0.1% Tween20 in 1xPBS, PFA, paraformaldehyde, UPR, unfolded protein response

## Abstract

Understanding how connective tissue cells respond to mechanical stimulation is important to human health and disease processes in musculoskeletal diseases. Injury to articular cartilage is a key risk factor in predisposition to tissue damage and degenerative osteoarthritis. Recently, we have discovered that mechanical injury to connective tissues including murine and porcine articular cartilage causes a significant increase in lysine-63 polyubiquitination. Here, we identified the ubiquitin signature that is unique to injured articular cartilage tissue upon mechanical injury (the “mechano-ubiquitinome”). A total of 463 ubiquitinated peptides were identified, with an enrichment of ubiquitinated peptides of proteins involved in protein processing in the endoplasmic reticulum (ER), also known as the ER-associated degradation response, including YOD1, BRCC3, ATXN3, and USP5 as well as the ER stress regulators, RAD23B, VCP/p97, and Ubiquilin 1. Enrichment of these proteins suggested an injury-induced ER stress response and, for instance, ER stress markers DDIT3/CHOP and BIP/GRP78 were upregulated following cartilage injury on the protein and gene expression levels. Similar ER stress induction was also observed in response to tail fin injury in zebrafish larvae, suggesting a generic response to tissue injury. Furthermore, a rapid increase in global DUB activity following injury and significant activity in human osteoarthritic cartilage was observed using DUB-specific activity probes. Combined, these results implicate the involvement of ubiquitination events and activation of a set of DUBs and ER stress regulators in cellular responses to cartilage tissue injury and in osteoarthritic cartilage tissues. This link through the ER-associated degradation pathway makes this protein set attractive for further investigation in *in vivo* models of tissue injury and for targeting in osteoarthritis and related musculoskeletal diseases.

## Highlights

• Articular cartilage rapidly responds to injury with a unique ubiquitin signature.
• Key ER stress regulators are significantly ubiquitinated upon mechanical injury.
• YOD1 and ATXN3 injury-ubiquitination status is linked to enhanced DUB activity.
• We observed a global DUB activation in human osteoarthritic cartilage samples.

## Graphical Abstract

**Figure:**
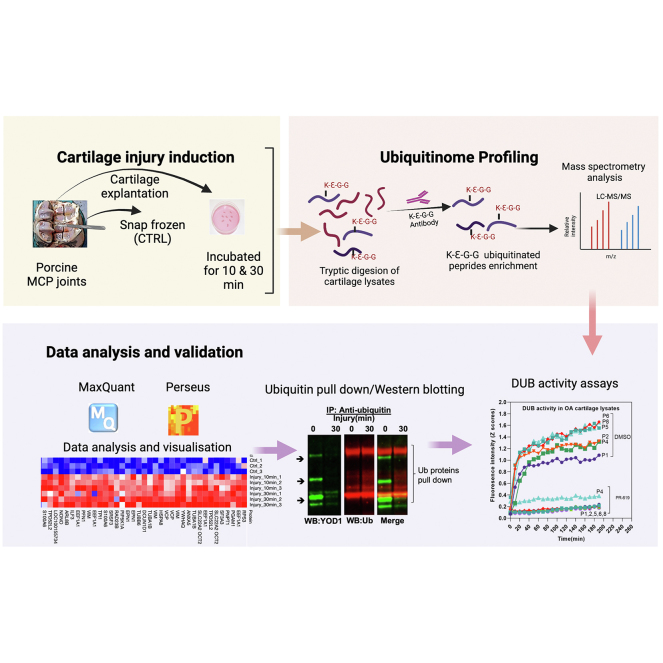

## In Brief

Using ubiquitomics, we identified a unique ubiquitin signature of articular cartilage upon mechanical injury. A set of deubiquitinases (DUBs) and ER stress regulators including YOD1, ATXN3, RAD23B, VCP/p97, and Ubiquilin 1 were differentially ubiquitinated and their ubiquitination status was linked to enhanced DUB activity and upregulated ER stress response following injury. We observed a rapid increase in global DUB activity in injured and in human osteoarthritic cartilage. Data implicates DUBs as potential targets to modulate injury-induced tissue damage in osteoarthritis.

Articulated joints guarantee the smooth movement of mammals. Articular cartilage serves as a cushion protecting the surfaces of bones in the joints by preventing friction and attrition. Mechanical injury to articular cartilage results in chronic inflammation, pain and predisposes to tissue damage, and loss of joint function. Progressive deterioration of the cartilage is the hallmark feature of osteoarthritis. Although multiple efforts have been made to understand how injury leads to osteoarthritis (1), it is still not fully understood how cells sense injury and what are the key cellular mechanism driving the damage.

Our previous work has shown that mechanical injury to articular cartilage activates within seconds the intracellular signaling pathways characteristic of the inflammatory response including TAK1 (2), NFkB, MAP kinases (2, 3, 4, 5), and Src, followed by induction of genes characteristic of inflammation (5). Cartilage is an avascular tissue, so connective tissue cells alone must directly sense the injury and respond with inflammatory signaling and gene induction. Interestingly, the response is not restricted to the injury site and propagates through the tissue with time (2). To identify the upstream mechanism, we have recently discovered that injury caused an overall increase in lysine-63 polyubiquitination in porcine and murine cartilage (2).

Protein ubiquitination is an upstream mechanism of posttranslational modifications which is emerging as a critical regulator of inflammatory signaling pathways (6). Ubiquitination is a reversible and dynamic reaction tightly controlled by the opposing actions of ubiquitin ligases and deubiquitinating enzymes (DUBs) (6). DUBs are cysteine and metalloproteases that work by reversing and editing ubiquitination in a specific manner and play several roles in ubiquitin homeostasis and negative regulation of ubiquitin signaling (Reviewed in (7)). DUB activity can be modulated by further posttranslational modifications including phosphorylation and ubiquitination. The presence of at least one ubiquitin-binding sites in DUBs facilitates their ubiquitination particularly when in complex with E3 ligases (Reviewed in (7)). Ubiquitination of DUBs could regulate their catalytic activity by either competing with binding of ubiquitinated substrates especially when associated with an E3 ligase or by enhancing activity through activating conformational changes (8, 9, 10).

At the cellular level, DUBs contribute to a plethora of cellular functions including cell signaling and modulation of endoplasmic reticulum (ER) stress. DUBs deregulation contributes to various disorders and age-related tissue degeneration (11, 12, 13). A number of DUBs are reported to control stress including YOD1 and ATXN3. YOD1 and ATXN3 are highly conserved DUBs that are involved in the regulation of the ER-associated degradation (ERAD) pathway by facilitating protein quality control through the ATPase VCP (14). YOD1 is recently reported to be involved in inflammation through interaction with the E3 ligase TRAF6 and regulation of the inflammatory cytokine IL1 through competing with the p62 NFkB axis (15). YOD1 attenuates neurogenic proteotoxicity through its deubiquitinating activity (16). The role of ATXN3 in neurodegeneration is also reported through regulation of ER stress (17, 18).

The ER is the site for biosynthesis for many cellular components. The ER controls crucial cellular functions, including protein synthesis, lipid synthesis, Ca^++^ storage and release, as well as Golgi and mitochondrial biogenesis. One of its key functions is protein quality control. Stress conditions that cause the protein import to exceed the protein folding capacity of ER trigger the unfolded protein response (UPR) (19). Failure of the ER to handle this stress response leads to cellular dysfunction, cell death, apoptosis, and diseases including neurodegeneration, diabetes mellitus, cancer, etc (Reviewed in (20, 21, 22)).

The UPR response is intended to restore the ER homeostasis through attenuation of protein translation and upregulation of ER-chaperone proteins CHOP, GRP78, and ATF3. Apoptosis is initiated when ER stress is not resolved (23, 24). GRP78 plays a key role in detection and transduction of ER UPR signaling to downstream transcription factors. GRP78 also known as BiP/HSPA5 is normally bound to the ER stress sensors exist in the transmembrane: IRE1, ATF6, and PERK. When misfolded proteins accumulate in ER lumen, GRP78 dissociates from these sensors initiating downstream signaling through these three arms (24, 25).

In cartilage, the UPR response plays a physiological role in both chondrogenesis and chondrocytes maturation during endochondral ossification (26). A prolonged ER stress response has also been reported to initiate chondrocyte death as identified in early onset osteoarthritis that is associated with several chondrodysplasias and collagenopathies characterized by prolonged UPR, ER stress, and ERAD. An extensive and a comprehensive review on the role of ER stress and UPR in cartilage and osteoarthritis is described by Hughes *et al.*, 2017 (26). Understanding how UPR signaling is regulated in cartilage injury and related cartilage pathophysiology is thus key to therapeutic targets discovery in osteoarthritis and related musculoskeletal diseases.

Here, we identify the unique ubiquitin signature of articular cartilage upon mechanical injury. We have observed differential ubiquitination patterns of YOD1 and ATXN3 DUBs that modulated their DUB activity in injured and osteoarthritic articular cartilage. We also report a significant modulation of the ubiquitination pattern of the key ER stress regulators: VCP, RAD23B, and Ubiquilin 1 as well as the two E2 enzymes UBE2N and UBE2L3. Our results highlight the importance of injury-induced ubiquitination events and DUB activation in regulating cellular responses that drive ER stress response. Those findings indicate that DUB activation contributes to the injury-induced inflammatory response and tissue damage, highlighting a possible mechanism for targeting in osteoarthritis.

## Experimental Procedures

### Cartilage *ex vivo* Injury Model and Preparation of Cartilage Tissue Lysates

Porcine articular cartilage was obtained from the metacarpophalangeal (MCP) joints of the pig forefeet or trotters. Pig trotters of freshly slaughtered 3- to 6-months-old pigs were purchased from a local farm. Trotters were first decontaminated in 2% Virkon and then equilibrated at 37 °C for 1 h before use. MCP joints were opened under sterile conditions, and injury to cartilage was induced by explantation from the articular surface as previously described (5). Cartilage was either snap-frozen (at time point 0) or cultured for indicated times in serum-free Dulbecco’s modified Eagle’s medium. An overview on the porcine cartilage injury model and the following experimental workflow is shown in supplemental Fig. S1.

### Preparation of Cartilage Tissue Lysates and Enrichment of Ubiquitinated Gly-Gly Peptides Using Immunoaffinity Purification

Cartilage tissues were explanted as described above from the articular surface of MCP joints of four pig trotters and pooled as one replicate for each time point. Cartilage tissues (4 g) for each replicate were lysed in 10 ml urea lysis buffer containing urea (9 M), Hepes buffer (20 mM) pH 8.0, sodium orthovanadate (1 mM), sodium pyrophosphate (2.5 mM), and β-glycerophosphate (1 mM) for 2 h at room temperature with continuous vortex mixing.

Tissue lysates were then sonicated three times using 15 W microtip for 15 s each. Samples were cooled on ice for 1 min between each sonication burst then cleared by centrifugation at 13,000 rpm for 15 min. Supernatants (20 mg of proteins/sample) were transferred to a new tube and then reduced with 1.25 mM DTT for 30 min at 55 °C. Extracts were then cooled down to room temperature and then alkylated using 1.9 mg/ml iodoacetamide for 15 min in the dark. Proteins were then digested overnight with trypsin at room temperature. Peptides were then purified using Sep-Pak C18 columns. Fifty micrograms of tryptic peptides were saved for global proteome analysis. Gly-Gly peptides were then purified using Ubiquitin remnant motif (K-ε-GG) kit from Cell Signalling Technology (5562). Tryptic peptides were incubated with K-ε-GG immunoaffinity beads for 2 h at 4 °C and then washed as per manufacturer’s instruction to remove unbound peptides. Gly-Gly enriched peptides were eluted using 0.15% TFA and then concentrated and purified for LC-tandem MS (MS/MS) analysis as per manufacture’s procedure.

### Mass Spectrometry Raw Data Processing, Visualization, and Pathway Analysis

Peptide material from pull-down experiments was resuspended in 20 μl water with 0.1% TFA and 2% acetonitrile. Samples were then injected into a Dionex Ultimate 3000 nano-ultra LC system (Thermo Fisher) coupled on-line to a Q Exactive mass spectrometer (Thermo Scientific) as described previously (27, 28). In brief, the samples were separated on an EASY-Spray LC column (PepMap RSLC C18, 500 mm × 75 μm ID, 2 μm particle size, Thermo Scientific) over 60 min for GlyGly immunoprecipitation and 120 min for the total proteome (gradient of 2–35% acetonitrile in 5% DMSO and 0.1% formic acid) at 250 nl/min. Acquisition of MS1 scans was performed at a resolution of 60,000 at 200 m/z. The most abundant precursor ions (top 12) were selected for HCD fragmentation.

#### Raw Data, Data Visualization, and Pathway Analysis

Mass spectrometry raw data were processed using MaxQuant software package (version 1.6.0.16). Default settings were used. MS/MS spectra were searched against the Uniprot Sus Scrofa (pig) proteome FASTA file (2018_49795 entries). Enzyme specificity was set to trypsin, and up to two missed cleavages were allowed for proteome analysis while three missed cleavages were allowed for ubiquitinome analysis. Variable modifications included diGly remnant of lysine[K(GG)] (+114.04 Da), methionine oxidation (+15.99 Da), and deamination of asparagines (N) and glutamines (Q) (+0.98 Da), while fixed modification was set as carbamidomethylation. Peptide precursor ions were searched with a maximum mass deviation of 4.5 ppm and fragment ions with a maximum mass deviation of 20 ppm. Peptide, protein, and site identifications were filtered at a false discovery rate (FDR) of 1% using the decoy database strategy. The minimal peptide length was seven amino acids, the minimum Andromeda score for modified peptides was 40, and the corresponding minimum delta score was 6 (default MaxQuant settings). diGly remnants of lysine were quantified by their extracted ion intensities (‘Intensity’ in MaxQuant).

Annotated spectra for all GlyGly-modified peptides were prepared in the freely available software MS-Viewer (https://msviewer.ucsf.edu/cgi-bin/msform.cgi?form=msviewer) using the msms.txt and all APL (.apl) files from MaxQuant output files according to instructions described on UCSF website (https://prospector.ucsf.edu/prospector/html/instruct/viewerman.htm#maxquant). The annotated spectra could be accessed through the Protein Prospector suite of software MS-Viewer using the search key iadtjnlpr2 or could be accessed directly at https://msviewer.ucsf.edu/prospector/cgi-bin/mssearch.cgi?report_title=MS-Viewer&search_key=iadtjnlpr2&search_name=msviewer. Zipped folder of APL files and the msms.text file are provided in Supplementary Data Folder (Supplementary folder_GlyGly Data for MS-Viewer) as peak list and results file for MS Viewer. All modified peptides and GlyGly(K) sites are described in supplemental Table S1.

Processed data was analyzed by label-free quantification and visualized using Perseus software package tools (v1.6.12.0) (29) (https://maxquant.net/perseus/) and Venny 2.1 (https://bioinfogp.cnb.csic.es/tools/venny/). Downstream analysis of the ‘proteinGroups.txt’ and ‘GlyGly. Txt’ output tables for proteome and enriched ubiquitinated peptides respectively were performed in Perseus. Columns for experiment and control set were selected and log transformed. Quantitative profiles were filtered for missing values and were then filtered independently for each of time point and control pairs, retaining only proteins and ubiquitin peptides that were quantified in all three replicates of either the injury time point or control pull-down. Missing values were imputed (width 0.3, down shift 1.8) before combining the tables and performing the multivolcano analysis. Data quality and normal distribution was inspected and represented in a multiscatter plot and histogram (supplemental Figs. S2 and S3).

Pathways and functional enrichment analysis of enriched hits and protein–protein interactions were performed using STRING v11.0 database (30). Heat maps were generated using the online platform Morpheus/Broad institute (https://software.broadinstitute.org/morpheus/).

### Validation of Enriched Ubiquitinated Proteins: Ubiquitin Enrichment Pull-Down Assays and Western Blotting

Cartilage was snap frozen immediately at the end of time points. Proteins were extracted from cartilage by lysis in two volumes of autoclaved glass beads lysis buffer (50 mM Tris, 5 mM MgCl2, 0.5 mM EDTA, and 250 mM sucrose) and one volume of acid washed glass beads (Sigma, G4649). One millimolar(1mM) DTT was added fresh to samples with vortex mixing at 4 °C for 2 to 3 h. Samples were then pelleted, clear supernatants were collected, and protein concentration was quantified using Qubit 4 fluorimeter as per manufacture’s procedure (Invitrogen). Ubiquitinated proteins were enriched from cleared tissue lysates (500 μg) using anti-ubiquitin antibody–conjugated beads (anti-mouse, Santa Cruz) overnight at 4 °C with rotation. Beads were pelleted and then washed with lysis buffer for five times/5 min each. Enriched proteins were then eluted by heating beads in 1× sample buffer. Further analysis of ubiquitinated proteins was performed by Western blotting using protein-specific antibodies. Protein A agarose beads were used as a negative control for nonspecific pull-down. Antibodies used in this analysis were from ProteinTech against VCP (10736-1-AP), Ubiquilin 1(23516-1-AP), UBE2N(10243-1-AP), UBE2L3(14415-1-AP), USP5 (10473-1-AP), RAD23B(12121-1-AP), YOD1(25370-1-AP), BRCC3(15391-1-AP), ATXN3(13505-1-AP), GRP78/BIP (11587-1-AP), DDIT3/CHOP (15204-1-AP), and β-actin (66009-1-Ig).

### DUB Activity Assays and Active DUBs Pull-down

#### Using Activity Probes

TAMRA-Ub-VME (UbiQ-050) and HA-Ahx-Ahx-Ub-VME (UbiQ-035) were purchased from UbiQ Bio BV. Porcine cartilage and human osteoarthritic cartilage tissue lysates were reacted with DUB activity probe for 45 min at 37 °C. Fifty to two hundreds fifty micrograms of proteins from lysates reacted with HA activity probe were then used for active DUBs pull-down using mouse anti-HA–conjugated magnetic Beads (88837, Thermo Scientific). Immunocomplexes were then washed three times using lysis buffer and then analyzed by Western blotting using rabbit anti-HA or DUB-specific antibodies. Lysates reacted with TAMRA probe were analyzed by SDS-PAGE followed by imaging fluorescence using Syngene gel imaging system.

#### Ubiquitin-Rhodamine (110)-Glycine Assay

DUB activity in cartilage tissue lysates were measured using ubiquitin-rhodamine (110)-glycine quenched substrate (Boston Biochem, cat No U-555). In the presence of active DUBs, the amide bond between the ubiquitin C-terminal glycine and rhodamine110Gly results in an increase in fluorescence at 535 nm (Exc. 485 nm). Ten to fifty micrograms of tissue lysates were incubated with 250 nM substrate, and fluorescence intensity reflecting activity was measured over time at 10 min intervals using a Varioskan LUX multimode microplate reader (Thermo Scientific). Readings were retrieved from ScanIT software (Thermo Scientific), and Z scores were calculated and plotted against time using GraphPad prism.

### Zebrafish Experiments

#### Husbandry and Maintenance

Adult Wild Type Zebrafish were maintained on a 14:10-h light/dark cycle at 28 °C in The Bateson Centre aquaria (University of Sheffield), under Animal Welfare and Ethical Review Bodies and UK Home Office–approved project license protocols.

#### Tail Fin Injury Assays

Tail fin injury was induced in zebrafish embryos by tail transection as described previously (31). Zebrafish larvae were anesthetized at three or five days post fertilization (dpf) by immersion in tricaine (0.168 mg/ml), and tail fin injury was induced by fin fold/tail transection using a sterile microscalpel. Embryos were then incubated in E3 medium at 28 °C as per indicated times in each experiment. Following incubation embryos were fixed for *in situ* hybridization and immunofluorescence.

#### Whole Mount *in Situ* Hybridization

Zebrafish GRP78/BIP and IL1 RNA probes were generated using PCR-based method and then DIG-labeled as described before (32). For whole mount *in situ* hybridization, larvae were fixed in 4% methanol-free paraformaldehyde (PFA) in PBST (0.1% Tween20 in 1xPBS) for 24 h at 4 °C with gentle rocking and then dehydrated in a methanol series (25%–100%) in PBS and stored at −20 °C overnight. Larvae were then rehydrated and then washed in PBST for four times (5 min each). Samples were digested with 10 μg/ml Proteinase K in PBST (Invitrogen). Larvae were washed briefly in PBST, refixed for 20 min in 4% PFA-PBST followed by washes in PBST, and then incubated at 67 °C for 2 h in prewarmed hybridization buffer. Hybridization buffer was replaced with probe buffer containing DIG-labeled RNA probes for either IL1 or GRP78 diluted in hybridization buffer and incubated at 67 °C overnight. Larvae were washed thoroughly at 67 °C with hybridization buffer, then washed in PBST, and incubated for 1 h in blocking buffer with gentle agitation. Larvae were incubated overnight at 4 °C with anti-DIG antibody in blocking buffer and then washed in PBST, followed by washes in staining wash solution. Larvae were then incubated in staining solution for 1 h and monitored for color development. Reactions were stopped with stop solution when appropriate color was achieved. Larvae were then fixed and dehydrated in methanol series as above, rehydrated then cleared with a series of glycerol dilution/H_2_O/0.1%Tween20. Stained larvae were stored in 75% glycerol:25% H_2_O.0.1% Tween20 at −20 °C until ready for imaging.

#### Whole Mount Immunofluorescence and Confocal Microscopy

Zebrafish larvae (3–5 dpf) were fixed in 4% PFA overnight at 4 °C. Fixed larvae were then washed three times in PBSTX buffer (0.1% triton X100 in 1xPBS) and then dehydrated in 100% methanol overnight at −20 °C. Following washing once in 1x PBSTX, larvae were then incubated in blocking buffer (PBSTX + 0.2% BSA + 5% serum) for 2 h at room temperature with gentle rocking. Larvae were incubated in primary antibodies (CHOP or GRP78) overnight at 4 °C in blocking solution at 1:300 dilutions. Antibody solution was then removed, and larvae were extensively washed with PBSTX at least six times for 10 min each before incubation with Alexa Flour 647–conjugated goat-anti rabbit secondary antibody overnight at 4 °C at 1:300 dilutions in blocking buffer. Samples were washed six times for 10 min each in PBSTX. Larvae were then transferred to imaging slides and arrayed as needed, and tails were cut to facilitate mounting using SlowFade Glass Soft-set Antifade Mountant with DAPI (Invitrogen, Catalog number S36920). Images of the tail regions were captured using the Nikon A1 confocal microscope (20×). Images were analyzed using FIJI software (NIH).

### Human Tissue Samples and Ethical Approval

Human osteoarthritic cartilage samples were obtained directly after knee replacement surgery from the South Yorkshire and North Derbyshire Musculoskeletal Biobank. Samples were collected with informed donor consent in full compliance with national and institutional ethical requirements (Sheffield REC 20/SC/0144), the United Kingdom Human Tissue Act, and the Declaration of Helsinki. Freshly collected samples were snap frozen immediately after surgery. Proteins were isolated from cartilage as described above; then lysates were used for DUB activity and pull-down assays as described above.

### RNA Extraction and RT-qPCR

RNA was isolated from articular cartilage as described previously (2). Briefly, cartilage tissues were homogenized in 1 ml Trizol (Invitrogen) for 1 min on ice and the homogenate was left at room temperature for 5 min and then mixed with 200 μl of BCIP solution. Samples were vortexed for 15 s and then incubated again at RT for 5 min followed by centrifugation at 13,000 rpm for 15 min at 4 °C. Aqueous layer was then separated and mixed with equal volume of 90% ethanol, then transferred to zymogen columns. RNA was bound to column filter followed by washing as per manufacture’s manual. DNA was digested on column using DNAse1 as per manufacture’s manual; then RNA was eluted in 20 μl ddH_2_O. Equal concentrations of RNA (1 μg) were used for reverse transcription reactions using superscript II reverse transcriptase for 2 h at 37 °C. Complementary DNA (cDNA) was then diluted and used in RT-PCR reaction using cyber green master mix (Applied Biosystems). RT-PCR reactions were performed in QuantStudio Flex 7 instrument, and data were analyzed using Max studio quantification. Cycle threshold (CT) values were used for calculations of ddCT and used for fold of change in gene expression compared to control and using GAPDH as housekeeping gene. Primers sequences are provided in supplemental Table S1.

### Experimental Design and Statistical Rational

The experiment series were designed to investigate the effect of mechanical tissue injury on modulating the articular cartilage ubiquitinome and proteome profile. We used a well-established ex vivo model of porcine cartilage injury as a source of cartilage tissue. Three time points (0, 10, 30 min) were investigated after injury to cover the early phase of cellular responses to injury where we observe a rapid induction of downstream inflammatory signaling pathways as we described previously (2, 3, 4, 5). For each time point, three biological replicates were analyzed to ensure reproducibility. Each biological replicate used 4 g of cartilage tissue which is pooled from four different porcine metacarpophalangeal joints. Snap-frozen tissue dissected cartilage was used as a control (three biological replicates). For each biological replicate, three technical replicates were analyzed using MS/MS. The choice of three replicates for each time point was based on previous initial work and on ensuring efficient handling of samples and quality of analysis. Proteome and ubiquitinome profiling was performed using reproducible label-free workflow (33). Raw mass spectrometry data were analyzed with MaxQuant, and downstream processing was performed using Perseus software (https://maxquant.net/perseus/). Protein peptide intensities were log transformed and mean normalized. The data sets are normally distributed. Comparison of injured and uninjured samples was performed using unpaired *t* test as a suitable statistical test for comparison of two groups. Exact *p* values are indicated in figures/heat maps as −log10pvalue. Statistics method is described in Figure legends where relevant.

**Fig. 1:**
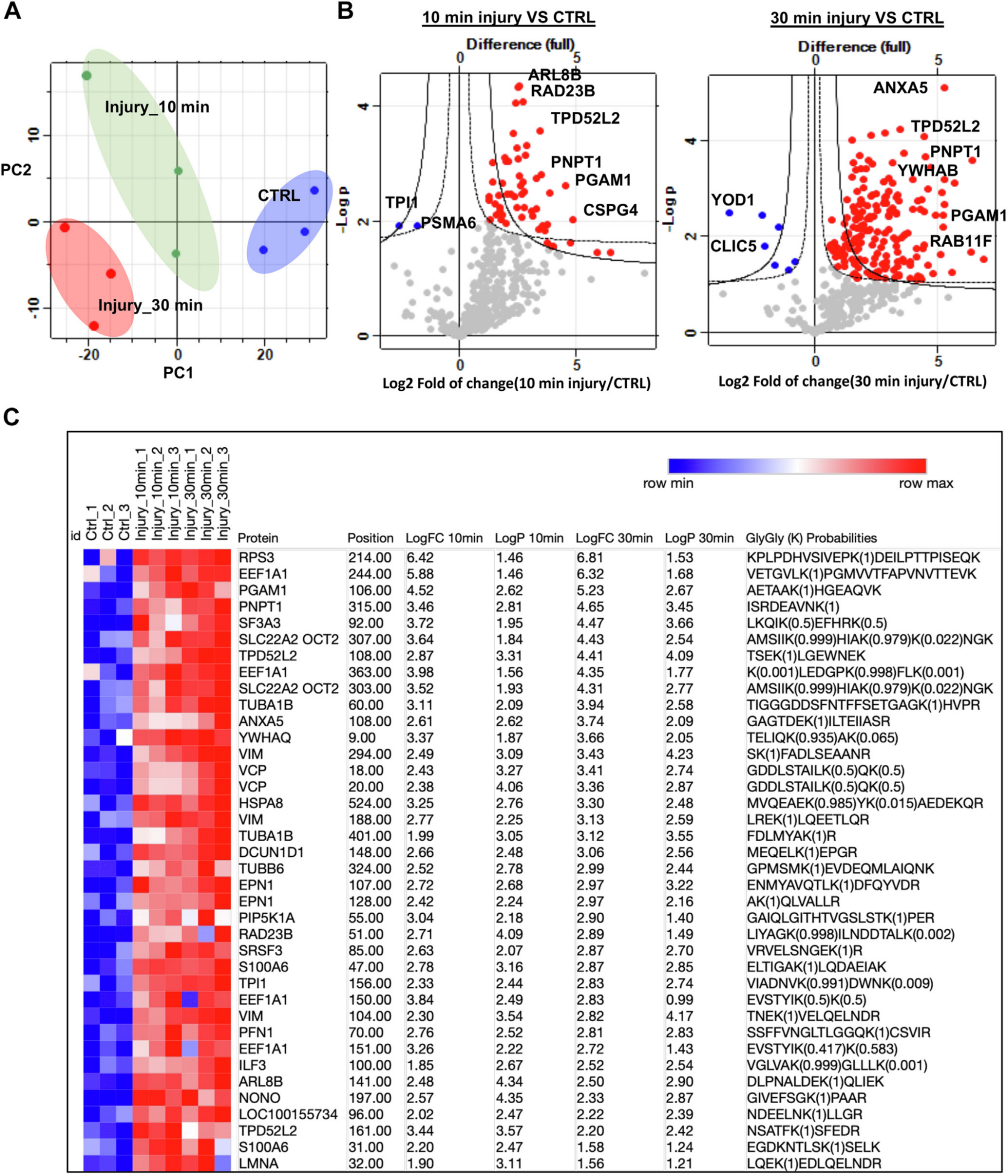
**The mechano-ubiquitinome of articular cartilage: ubiquitomics landscape of articular cartilage before and after mechanical injury.***A*, principal component analysis of ubiquitinated peptides intensity in control uninjured (CTRL) and injured articular cartilage samples 10 and 30 min after injury reveals strong clustering of samples by injury response in the three biological replicates. *B*, volcano plot representation of ubiquitomics analysis of articular cartilage samples before (CTRL) and after injury (10 min and 30 min). *C*, heat map of differentially ubiquitinated peptides in response to injury that are commonly detected after 10 and 30 min compared to uninjured control ranked by log2 fold of change (LogFC) at 30 min after injury. Statistics by unpaired *t* test.

**Fig. 2:**
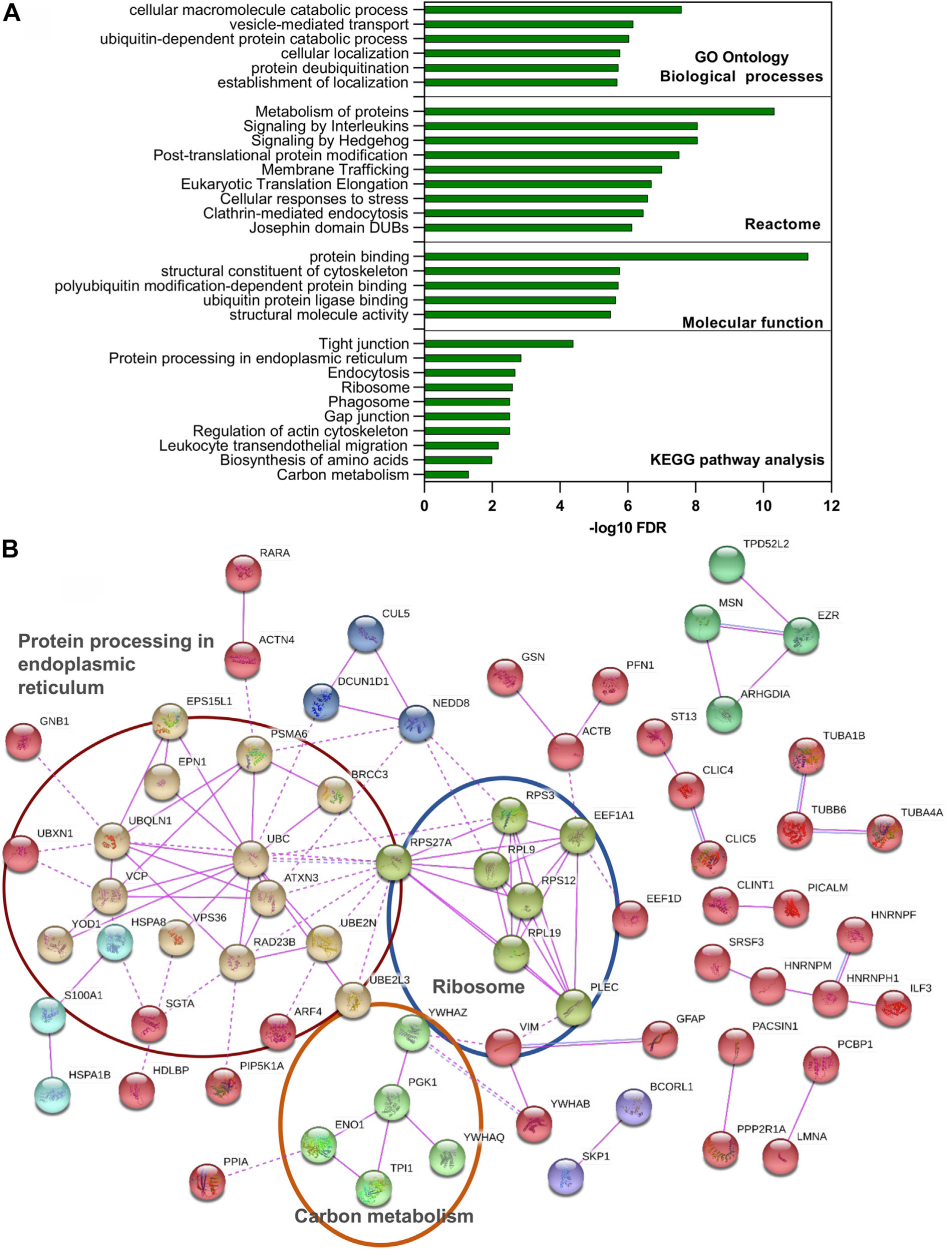
**Protein network and enriched pathway analysis of differentially ubiquitinated proteins in articular cartilageafterinjury.***A*, pathway analysis of enriched ubiquitinated proteins modulated in response to injury using KEGG, Reactome, and Go ontology pathway analysis databases. *B*, ubiquitinated peptides identified in cartilage after injury were mapped to proteins and were further analyzed for direct protein–protein interactions using STRING database. Disconnected nodes are hidden and only connections based on experimental evidence in STRING are shown. Colors present proteins in the same cluster. *Solid lines* represent protein-protein associations in the same cluster. *Dotted lines* represent protein associations at the edges between clusters.

**Fig. 3:**
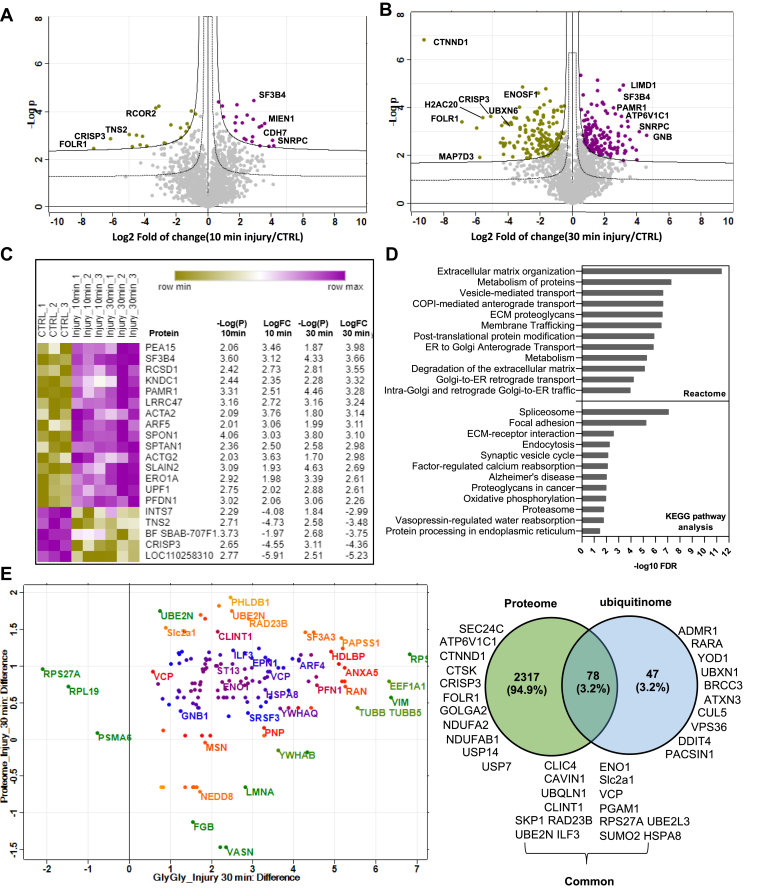
**Proteome analysis of porcine articular cartilage before and after injury and comparison with ubiquitinome analysis.***A* and *B*, volcano plots representation of proteomics analysis of articular cartilage samples before (CTRL) and after injury (10 min and 30 min). *C*, heat map of top 20 proteins differentially regulated after injury that are commonly detected after 10 and 30 min are shown and ranked by log2 fold of change in the three biological replicates. Statistics by unpaired *t* test. *D*, KEGG and Reactome pathway analysis of enriched proteins in proteome analysis after injury using STRING database. *E*, cross analysis of cartilage ubiquitinome and proteome. Scatter plot shows proteins that are commonly detected in ubiquitinome and proteome 30 min after injury. Venn diagram shows a comparison of significantly enriched ubiquitinated proteins and total proteome 30 min after injury. Difference: Log2 fold of change.

**Fig. 4:**
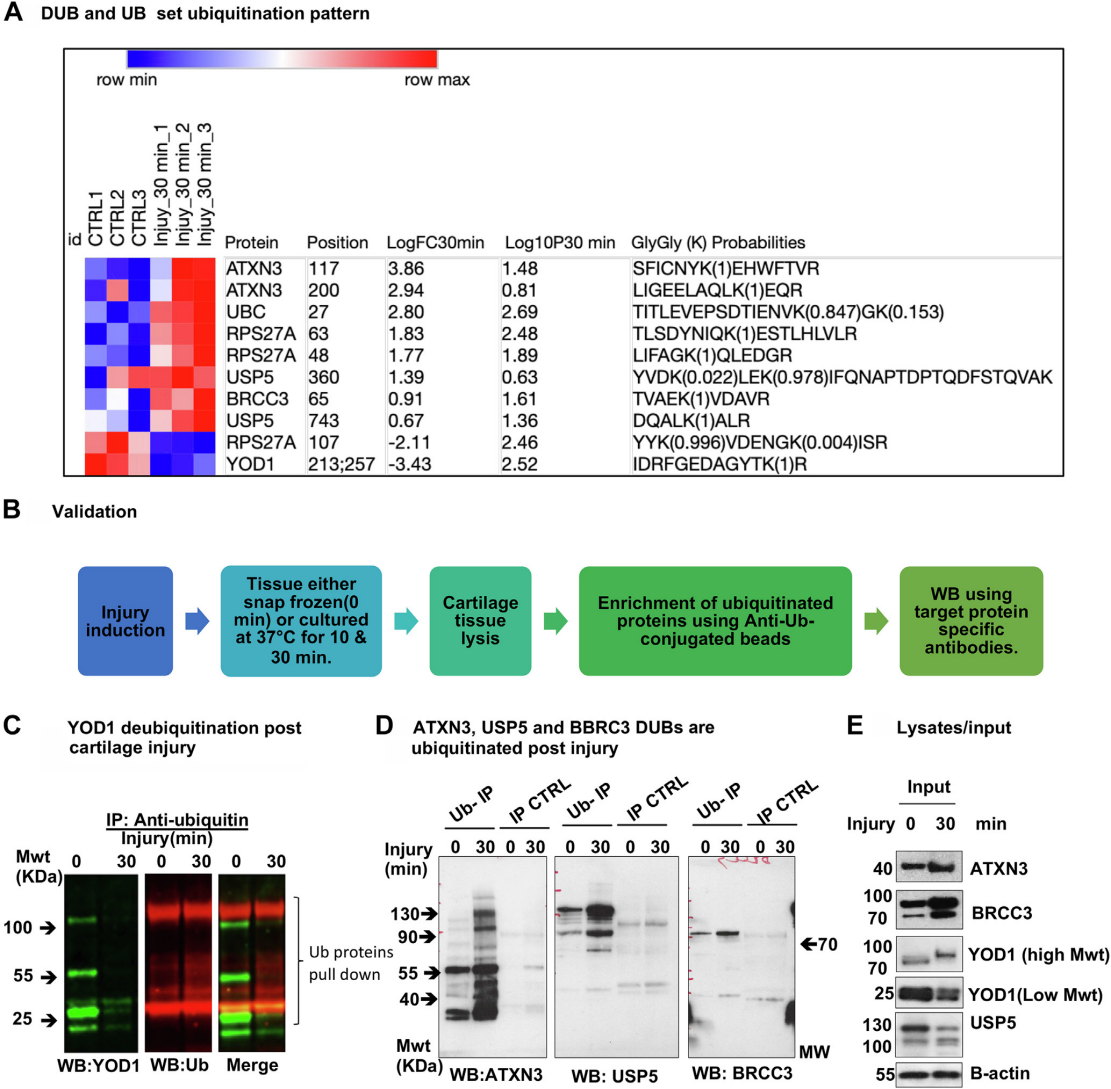
**A group of ER-associated DUBs are differentially ubiquitinated in response to articular cartilage injury.***A*, heat map of differentially ubiquitinated peptides of DUB proteins in response to injury (30 min) compared to uninjured control ranked by log2 fold of change(LogFC30 min) in three biological replicates. Statistics by unpaired *t* test. *B*, a summary of the validation approach used to confirm ubiquitomics analysis. *C*–*E*, porcine cartilage was explanted to induce injury and either snap frozen(0 min) or cultured at 37 °C for 30 min. Cartilage was then lysed as described in Experimental Procedures. Ubiquitinated proteins were enriched using anti-ubiquitin–conjugated agarose beads (Ub-IP) and agarose beads were used as a control (IP CTRL). After immunoprecipitation, immunocomplexes were analyzed by Western blotting using target protein–specific antibodies for DUB proteins in (*C*) YOD1 and (*D*) ATXN3, USP5, and BRCC3. *Arrows* indicate the enriched specific proteins. Shown are the blots representative of three biological replicates. *E*, protein levels of candidate proteins were analyzed in input tissue lysates samples. *β*-actin was used as an internal loading control. Numbers on blots reflect protein molecular weight. DUB, deubiquitinase; ER, endoplasmic reticulum.

**Fig. 5:**
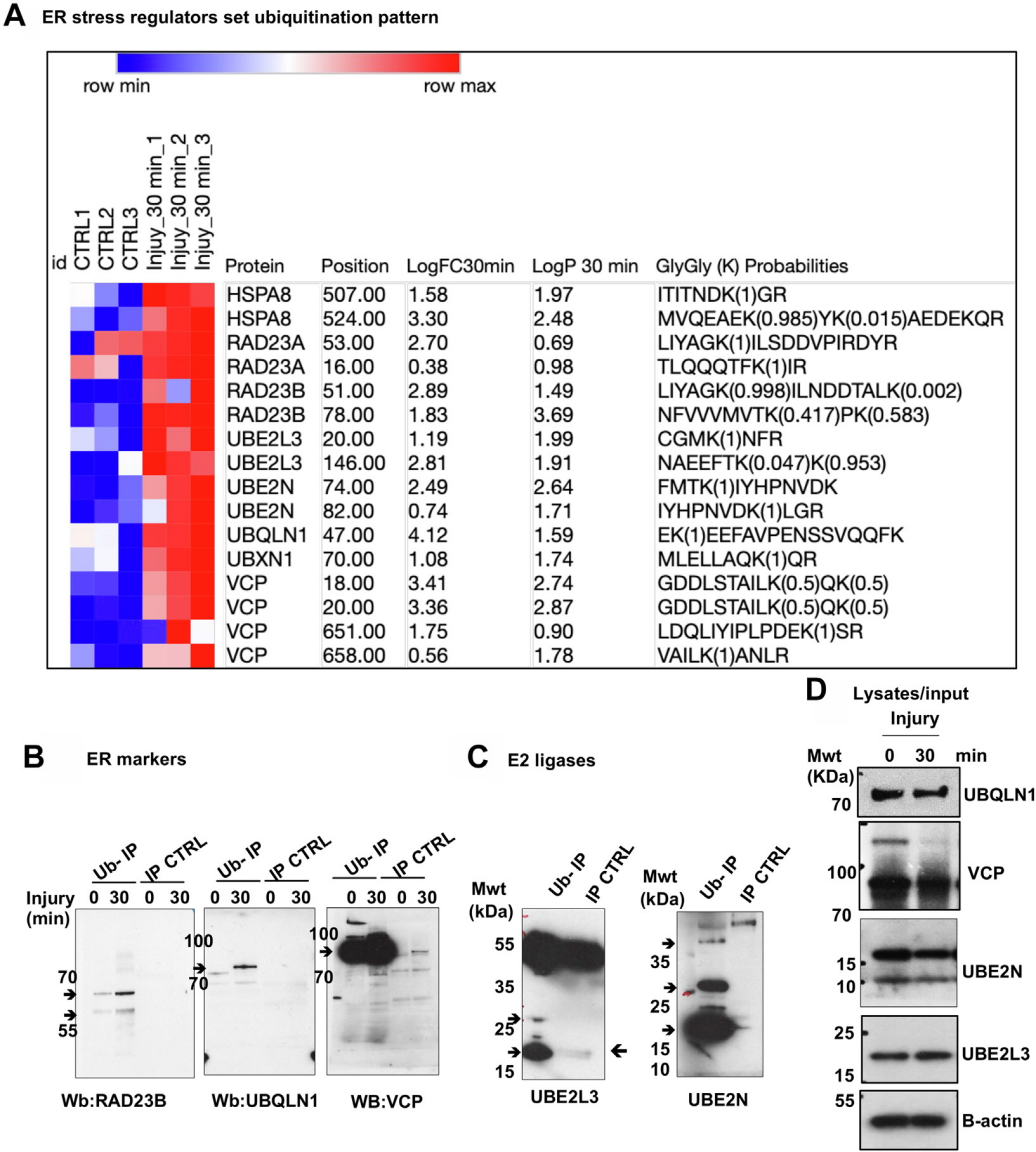
**A group of ER stress regulators are differentially ubiquitinated in response to articular cartilage injury.***A*, heat map of differentially ubiquitinated peptides of ERAD set proteins including ER stress regulators and E2 ligases in response to injury (30 min) compared to uninjured control ranked by log2 fold of change in three biological replicates. Statistics by unpaired *t* test. Validation of ubiquitinated proteins was done as described in Experimental Procedures and Figure 4*B* to confirm ubiquitomics analysis. *B*–*D*, porcine cartilage was explanted to induce injury and either snap frozen(0 min) or cultured at 37 °C for 30 min. Cartilage was then lysed as described in Experimental Procedures. Ubiquitinated proteins were enriched using anti-ubiquitin–conjugated agarose beads (Ub-IP) and agarose beads were used as a control (IP CTRL). After immunoprecipitation, immunocomplexes were analyzed by Western blotting using target protein–specific antibodies for (*B*) ER stress markers (VCP, RAD23B, UBQLN1), or (*C*) E2 enzymes UBE2L3 or UBE2N. *Arrows* indicate the enriched specific proteins. Shown are the blots representative of three biological replicates. *D*, protein level of candidate proteins were analyzed in input tissue lysate samples. *β*-actin was used as an internal loading control. Numbers on blots reflect protein molecular weight. ER, endoplasmic reticulum.

Groups were described as per treatment or based on injury status. Human subjects were labeled with a donor number. Number of biological replicates were indicated for ubiquitomics and proteomics. Samples from different individual human donors are representing different replicates.

**Fig. 6:**
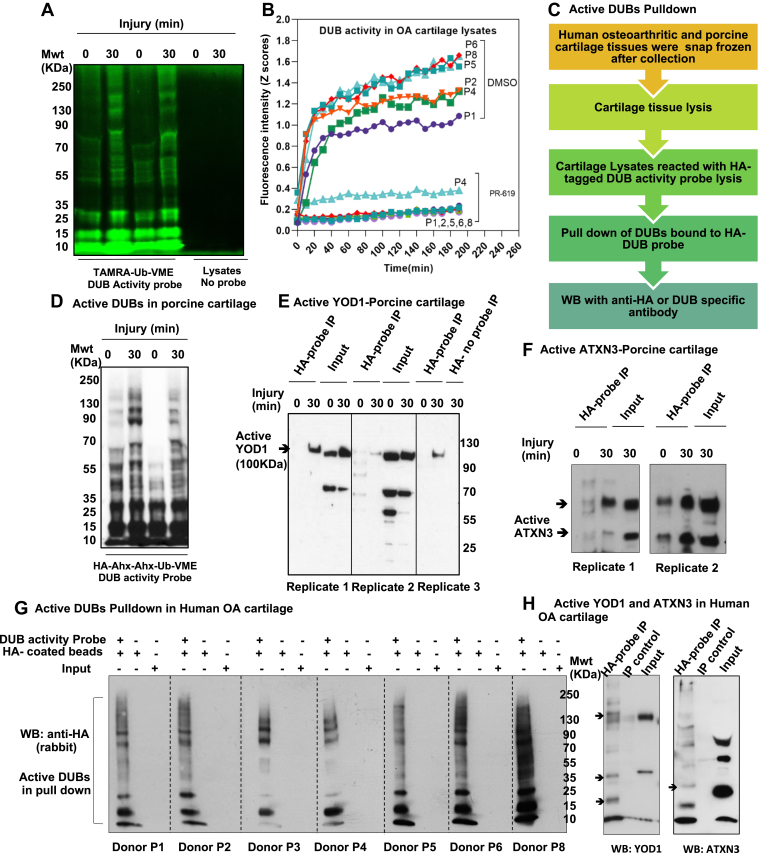
**Global DUB activity is detected in injured and osteoarthritic cartilage with YOD1 and ATXN3 DUBs enriched in active DUBs pull-down.***A*, porcine articular cartilage lysates from control and injured samples were incubated with the DUB activity probe TAMRA-Ub-VME for 30 min at 37 °C. In-gel fluorescence scan of TAMRA-Ub-VME DUB activity probed reactions were obtained with a XX6 gel imaging system (Syngene) (resolution = 100 μm and exposure time of 60 s, λex/λem = 550/590 nm). *B*, UBRH110 DUB activity assay of human OA cartilage lysates in the presence or absence of PR-619 DUB inhibitor. Fifty micrograms of tissue lysates were reacted with the UBRH110 substrate and activity of the reacted DUBs was immediately detected by emitted fluorescence at 535 nm at 10 min intervals using Varioskan microplate reader. *C*, flow chart of active DUBs pull-down assay. *D*, pull-down of active DUBs in porcine cartilage lysates before and after injury using HA-Ahx-Ahx-Ub-VME DUB activity probed reactions of cartilage. Fifty micrograms of porcine cartilage lysates were reacted with HA-Ahx-Ahx-Ub-VME DUB activity probe for 45 min at 37 °C and then subjected to Western blotting using anti-HA antibody. Porcine cartilage (*E* and *F*) and human OA cartilage (*G* and *H*) obtained and lysed as described in Experimental Procedures. Two hundreds and fifty micrograms of lysates were reacted with the DUB activity probe HA-Ahx-Ahx-Ub-VME for 45 min at 37 °C, then subjected to immunoprecipitation of active DUBs using mouse anti-HA antibody–conjugated magnetic beads. Immunocomplexes were analyzed by Western blotting and compared with input and IP control using DUB specific antibodies YOD1 (*E* and *H*), ATXN3 (*F* and *H*). *G*, active DUBs pull-down in OA cartilage tissue lysates from seven different donors were analyzed by Western blotting using rabbit anti-HA antibody. Numbers on blots reflect protein molecular weight. DUB, deubiquitinase.

## Results

### Mechanical Injury to Articular Cartilage Significantly Modulates the Cartilage Ubiquitinome and Proteome Profiles

We previously showed that injury to murine and porcine articular cartilage causes a significant increase in K63- polyubiquitination (2). To identify the protein substrates that are differentially ubiquitinated in response to mechanical injury (the “mechano-ubiquitinome ”), we used ubiquitin remnant motif analysis (34) of uninjured *versus* injured porcine articular cartilage. Based on our previous work, it was identified that the changes in ubiquitination following mechanical injury are rapid and transient, with an optimum increase in ubiquitination at 30 min (2). To identify the rapidly and differentially ubiquitinated peptides in response to articular cartilage injury, porcine cartilage was injured, and biological triplicate samples were collected at 0, 10, and 30 min following injury. Injured tissues were lysed in a urea-containing buffer and subjected to tryptic digestion. Ubiquitinated peptides were enriched from digested tissue lysates using anti-K-ε-GG antibody as described in Experimental Procedures. Samples were then analyzed by LC–MS/MS for quantitative profiles of nonredundant enrichment of ubiquitinated sequences/peptides as well as global proteome (Experimental setup in supplemental Fig. S1) and as previously described (35). All modified peptides and GlyGly(K) sites are described in supplemental Table S2.

Four hundred sixty-three (463) ubiquitinated peptides were detected. Using Perseus Software, data were filtered based on valid values and imputed missing values from normal distribution resulting in 408 ubiquitinated peptides (supplemental Table S3). Principal component analysis of control and injured time sets showed a good separation between samples based on injury stimulation (Fig. 1*A*). Out of the 408 enriched ubiquitinated peptides, 201 with three charges and 189 with two charges, and 18 peptides with four charges were identified. Two different cut-off parameters were used to represent significantly enriched peptides after injury. Class A for FDR 0.05 and S0 0.5 and Class B for FDR 0.05 and S0 0.1. Volcano plots in Figure 1*B* show that a total of 170 ubiquitinated peptides were differentially enriched following 30 min after injury (Class A) and 200 enriched peptides (Class B) compared to 39 ubiquitinated peptides were enriched in the 10 min injury data set (Class A), while 52 ubiquitinated peptides were enriched in Class B (supplemental Table S3). The heat map in Figure 1*C* shows a group of significantly enriched ubiquitinated peptides that are common to both the 10 min and 30 min injury data sets, ranked by log2 fold of change in the 30 min-injury set. This group included 38 peptides mapped to 27 proteins for example RPS3, VCP, HSPA8, and ARL8.

These results highlight that injury to articular cartilage causes a rapid change in protein ubiquitination patterns of a specific set of cellular proteins. This change occurs very rapidly within 10 min and is optimum at 30 min.

The enriched ubiquitinated peptides after injury were mapped to 115 proteins. Enriched pathways were detected using STRING v11.0 database (30). Examples of the top enriched pathways are shown in Figure 2*A* bar chart. The top enriched KEGG pathways included Tight junction (9 proteins), ‘’Protein processing in endoplasmic reticulum’’ (7 proteins), Endocytosis (6 proteins), biosynthesis of amino acids (5 proteins), glycolysis/gluconeogenesis (4 proteins), and Ribosome (5 proteins) and carbon metabolism (5 proteins). Reactome pathways analysis showed a significant enrichment of vesicle-mediated transport (20 proteins), membrane trafficking, clathrin-mediated endocytosis, translocation of GLUT4 to membrane and Josephin domain DUBs (5 proteins), and metabolism of proteins. Details of the enriched pathways and linked proteins are shown in supplemental Table S4. Mapped proteins were further analyzed for protein–protein interactions based on experimental evidence using STRING database. Disconnected nodes were excluded and only directly interacting proteins based on experimental evidence are shown. Out of 115 mapped proteins, 68 proteins showed node connections based on experimental evidence with PPI enrichment *p*-value = 2.2e-05. Those were separated into five main clusters based on protein function including ERAD, carbon metabolism, and ribosome protein clusters (Fig. 2*B*).

Global proteome of cartilage lysates before and after injury was also analyzed using mass spectrometric analysis as described in Experimental Procedures. Two thousand four hundred fifty (2450) proteins were detected across all samples (supplemental Table S5). Using Perseus Software, data were filtered based on valid values and imputed missing values from normal distribution, resulting in 2394 annotated proteins. Class A significance (S0 0.5 & FDR 0.01) of proteome showed enrichment of 564 proteins in cartilage at 30 min following injury compared to 63 proteins (Class A), which were enriched in the 10 min proteome data set. Class B significance (S0 0.5 & FDR 0.05) of proteome showed 1145 proteins enriched at 30 min while 201 proteins were enriched 10 min after injury (Volcano plots in Fig. 3, *A* and *B*). Heat map in Figure 3*C* shows the top 20 proteins that are commonly modulated after injury after 10 and 30 min. Enriched proteins (Class A) after 30 min injury were interrogated using the STRING database to identify protein–protein interaction and enriched pathways. The bar chart in Figure 3*D* shows examples of enriched KEGG and Reactome pathways, including extracellular matrix organization, protein metabolism, and protein processing in ER (UGGT1, UBXN6, HSPA6, UBE2G2, SEC24C, RAD23B, ERO1L, DNAJB11, BAG1, PRKCSH and RAD23A).

Cross analysis of the injury proteome and injury ubiquitinome showed that, of the 115 proteins that are significantly ubiquitinated upon injury, 78 proteins were also enriched after injury in the proteome data set (3.2% of total proteome) (Fig. 3*E* and supplemental Table S6) while 34 proteins were uniquely enriched in ubiquitinome data set after injury including YOD1, ADMR1, UBXN1, ATXN3, and DDIT4 proteins.

### Cartilage Injury Causes Differential Ubiquitination of a Protein Subset of ER Regulators and DUBs Involved in the Regulation of the ERAD Response

We interrogated the set of differentially ubiquitinated peptides after injury using STRING database and identified a cluster of enriched peptides that are highly connected in a protein network. The proteins identified in this cluster were mapped to the GO term “response to cellular stress” and the KEGG pathway “protein processing in the endoplasmic reticulum” also known as the ERAD response. This cluster was also mapped to the GO terms ‘’ubiquitin protein ligase binding’’ and ‘’protein deubiquitination’’ (Fig. 2). This protein cluster comprised the three DUBs YOD1, BRCC3, and ATXN3, the two E2 ubiquitin ligases UBE2N and UBE2L3, as well as the ER stress regulators VCP/p97, RAD23B, Ubiquilin 1, UBXN1, SAR1A, and HSPA8 proteins (Fig. 2*B*). Hereafter, this set will be referred to as the ER/DUB protein set. The identified ER/DUB set of proteins is well known for their involvement in the ERAD response (36, 37, 38), making this protein set attractive for further analysis.

The heat map in Figure 4*A* shows the ubiquitination pattern of the four DUBs (YOD1, ATXN3, USP5, and BBRC3) after 30 min of injury in all replicates compared to uninjured controls. We also observed enrichment of ubiquitin protein peptides at lysine 48 and lysine 63 upon injury which confirms our previously reported observations (2). We have validated and confirmed the differential ubiquitination of these DUBs in response to injury using ubiquitin enrichment pull-down assays followed by Western blotting as described in Experimental Procedures and in Figure 4*B*. YOD1 is enriched in ubiquitinated fraction in uninjured controls and was significantly deubiquitinated after injury (Fig. 4*C*). The three DUBs BRCC3, ATXN3, and USP5 ubiquitination is enhanced following injury (Fig. 4*D*). Figure 4*E* shows the protein levels of this DUB set in input tissue lysates before and after injury for the samples used in ubiquitin enrichment assays compared to β-actin as a loading control. Examples of annotated spectra for GlyGly(K) peptide of UBC K63, UBC K48, and YOD1 are shown in supplemental Fig. S4.

The heat map in Figure 5*A* shows the ubiquitination pattern of a group of ER stress regulators proteins following 30 min of injury in all replicates compared to uninjured controls. We have validated and confirmed the differential ubiquitination of some of these proteins in response to injury using ubiquitin enrichment pull-down assays followed by Western blotting as described in Experimental Procedures. Figure 5*B* shows the enrichment of the three ER stress regulators VCP, ubiquilin 1, and RAD23B in ubiquitinated proteins pull-down 30 min after injury compared to the uninjured control. We also validated the ubiquitination of the two E2 ubiquitin-conjugating enzymes UBE2L3 and UBE2N as shown in Figure 5*C*. Figure 5*D* shows the protein levels of this ER protein set in input tissue lysates before and after injury used for ubiquitin enrichment assay compared to β-actin as a loading control.

These results validate the ubiquitomics mass spectrometry analysis and confirm the differential ubiquitination of the ER/DUB protein set after injury. Data shown here suggests a significant involvement of ER stress response and DUB-related proteins in cellular responses to mechanical injury to articular cartilage.

### A Rapid Increase in Global DUB Activity Following Injury to Porcine Cartilage Tissue and Detection of Significant DUB Activity in Human Osteoarthritic Cartilage with YOD1 and ATXN3 DUBs Enriched in Active DUBs Pull-Down

To investigate if DUBs are active in injured or diseased cartilage, we have analyzed the global DUB activity in porcine and human articular cartilage. Using DUB-specific activity probes, we compared the DUB activity in injured and control porcine articular cartilage samples. Interestingly, we observed an increase in DUB activity following injury as shown in Figure 6*A*. At the same time, other DUBs appear to be enriched in the control set compared to the injury set, indicating possible deactivation of another group of DUBs upon injury.

To quantify the DUBs activity in human OA cartilage tissue lysates, we also used a quenched ubiquitin Rhodamine 110 substrate. DUB activity was detected in tissue lysates in osteoarthritic cartilage of six donors. The substrate fluorescence intensity increased over time reflecting enhanced DUBs activity (Fig. 6*B*). Pre-incubation of tissue lysates with PR-619 inhibitor abolished this activity confirming the activity we detected is related to active DUBs in the lysates (Fig. 6B).

As described above, we have observed a differential ubiquitination pattern of YOD1 (lysine 213) and ATXN3 (lysine 117 and lysine 200) in response to cartilage injury (Fig. 4*C*). Ubiquitination of ATXN3 at lysine 200 and YOD1 at lysine 213/257 have not been reported before. It is well described that DUB activity can itself be modulated by ubiquitination (8, 9, 10). This implicates a possible modulation of the enzymatic activity of these DUBs in our experiments following cartilage injury. To analyze if differential ubiquitination of these identified DUBs in response to injury modulates their protease activity, we used DUB activity–specific probed reactions, with active DUBs pulled down and analyzed by Western blotting using DUB-specific antibodies as shown in flow chart in Figure 6*C*. Briefly, porcine cartilage tissue lysates (before and after injury) were reacted with HA-tagged DUB activity probe (HA-Ahx-Ahx-Ub-VME) for 45 min at 37 °C. Reactions were then stopped and active DUBs that bind to the activity probe were pulled down using mouse HA antibodies. Pull-down complexes were then analyzed by Western blotting using rabbit anti-HA– (Fig. 6*D*), anti-YOD1– (Fig. 6*E*), anti-ATXN3– (Fig. 6*F*), and anti-USP5– (supplemental Fig. S5) specific antibodies. We observed that YOD1 and ATXN3 were pulled down in active DUB fractions following injury (Fig. 6, E–F), implicating that injury-modulated ubiquitination of these DUBs enhances their protease activity. We observed USP5 activation after injury; however, USP5 blot showed a nonspecific band above the predicted size of UPS5 but also showed enrichment of active USP5 in the pull-down compared to IP control (supplemental Fig. S5).

To confirm disease relevance of these findings, we investigated the DUB activity in tissue lysates of human OA cartilage using DUB activity assays (as described above). Osteoarthritic cartilage lysates from seven human donors were reacted with DUB activity probes as described in Experimental Procedures, and samples were treated as described for porcine cartilage. Western blotting of HA pull-down showed an enrichment of active DUBs in tissue lysates-activity probe immunoprecipitated (probe-IP) samples compared to beads control (CTRL-IP) indicating a significant level of DUB activity in those samples (Fig. 6*G*). YOD1 and ATXN3 were detected in active DUBs pull-down fraction compared to control (Fig. 6*H*) indicating that they are activated in osteoarthritic cartilage. These findings demonstrate a global activation of DUBs in osteoarthritic and injured articular cartilage and enrichment of active ATXN3 and YOD1.

### Injury to Articular Cartilage and Zebrafish Embryos Causes Induction of an ER Stress Response

**Fig. 7:**
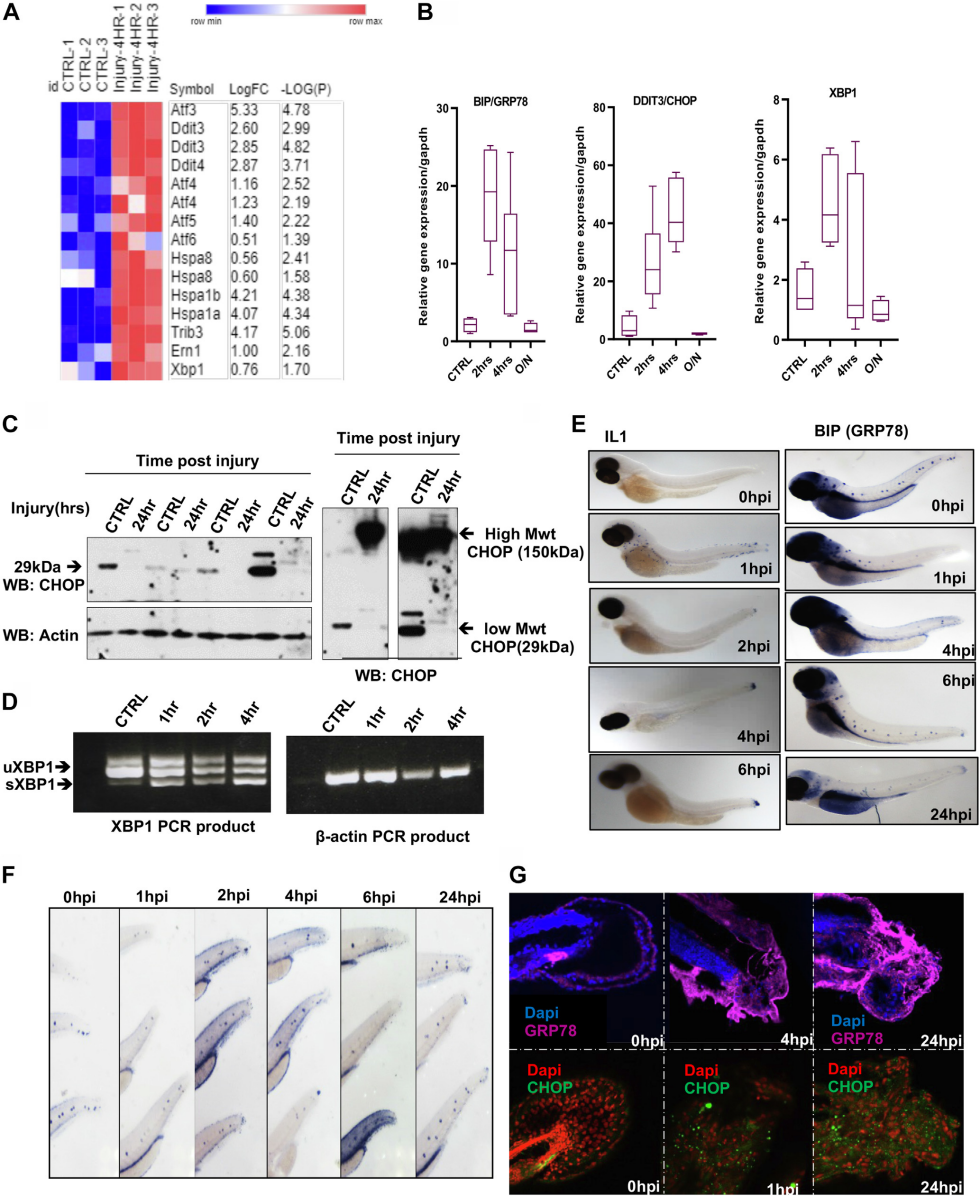
**Injury upregulates the ER stress markers in murine and porcine articular cartilage as well as in zebrafish embryos.***A*, heat map of ER stress genes in murine hip cartilage before and after injury. ER stress genes expression levels were retrieved from our previously published murine hip injury microarray data set (GEO: GSE155892). Statistics by unpaired t test. *B*, RT-qPCR of BIP, DDIT3, and XBP1 genes in porcine articular cartilage upon injury. *C*, CHOP protein levels in porcine cartilage lysates before and after 24 h after injury (four biological replicates). *D*, PCR products of XBP1 from porcine cartilage before and after injury were analyzed by agarose gel electrophoresis. uXBP1:unspliced XBP1 and sXBP1: spliced XBP1. *E*, *in situ* hybridization of IL1 (positive control) and GRP78 RNA probes in 3dpf zebrafish embryos before and after tail fin injury. Images were obtained using Stereomicroscope-integrated colored camera. *F*, GRP78 RNA staining in zebrafish tails before and after injury (*purple stain*). *G*, immunofluorescence analysis of GRP78 and CHOP proteins in zebrafish injury. DAPI for molecular stain. Images z stacks were obtained using A1 Nikon confocal microscope and are shown after maximum intensity projection. ER, endoplasmic reticulum.

Having observed that articular cartilage injury induces ubiquitination of the key ER stress regulators VCP, Ubiquilin 1, and RAD23B (Fig. 5*B*), we hypothesized this may reflect the induction of ER stress response upon mechanical injury. To test the direct link between cartilage injury and ER stress, we interrogated our previously published microarray data of murine hip cartilage before and after 4 h of tissue injury (GEO accession number: GSE155892) for gene expression levels of ER stress genes. We observed a significant upregulation in the expression levels of ER stress–related mouse genes including Atf3, Atf4, Atf5, Ddit4, Trib3, Hspa1a &b, and Ddit3/CHOP. A marginal increase was observed in the levels of Xbp1, HSPA8, and Ern1 genes upon injury (Fig. 7*A*). We then investigated the gene and protein expression of ER stress markers BIP/GRP78, XBP1, and DDIT3/CHOP following injury to porcine articular cartilage. Porcine cartilage tissue was injured as for the indicated times and then processed either for RNA or protein extraction as described in Experimental Procedures. Gene expression levels of CHOP, GRP78, and XBP1 ER stress genes were detected using real time RT-qPCR. GAPDH was used as housekeeping gene. Figure 7*B* shows a transient elevation of ER stress genes expression following mechanical injury with a peak of induction between 2 and 4 h. This increase reset to basal levels by overnight incubation of cartilage following injury. Figure 7*C* shows differential protein levels of CHOP 24 h following cartilage injury. Interestingly, we have noticed that injury lowered expression levels of CHOP lower molecular weight form (27 kDa) and induced a higher molecular weight form after 24 h of injury. This may be a supershift form of CHOP protein caused by its ubiquitination and indicating its activity. Ubiquitination of CHOP protein causes its shift to 50 kDa was previously reported (39). Induction of Xbp-1 gene expression as well as its splicing is also a marker of ER stress activation (40). We have noticed that Xbp-1–spliced variant is induced following tissue injury in articular cartilage as early as 1 h after injury (Fig. 7*D*).

Our previous work showed that injury responses are generic to connective tissues in porcine, murine, and zebrafish tissues ((2, 41, 42) & unpublished data). To test if injury caused an induction of ER stress response *in vivo*, we have used a well-established tail fin injury model of developing zebrafish embryos. Injury was induced by transection of zebrafish embryo tails at 3 days post fertilization (3dpf) as described in Experimental Procedures. We evaluated the induction of ER stress by increased levels of GRP78 mRNA and GRP78 and CHOP proteins at the injury site using RNA *in situ* hybridization and immunofluorescence, respectively (Fig. 7, *E-*G). We have observed that GRP78 expression in uninjured control embryos is localized around neuromasts, while expression levels are increased around the injury site as early as 2 h after injury. IL1 RNA probe was used as a positive control for injury responses in zebrafish embryos (Fig. 7*E* left panel). We also observed increased protein levels of CHOP and GRP78 at the tail fin injury site using immunofluorescence (Fig. 7*G*).

Taken together, these data indicate that mechanical injury to articular cartilage directly causes an induced ER stress response reflected by increased gene and protein expression levels of ER stress markers. The data also indicates that the responses to tissue injury are generic and the effect on ER stress is also observed *in vivo* as well as *ex vivo* models of tissue injury.

To investigate if the expression pattern of ER stress responsive genes is modulated in osteoarthritis, we interrogated the publicly available Gene Expression Omnibus RNA-seq data available for osteoarthritic tissue samples (synovium and cartilage) (supplemental Fig. S6*A*). Interestingly, we have observed a significant reduction in the expression of ER transcription factors DDIT3/CHOP, ATF3, XBP1 while ATF6 and ATF4 transcription factors levels were not changed. ER stress genes HSPA5 and ERN1 were also significantly reduced in OA cartilage compared to normal. DERL1,2, and 3 genes were not changed in diseased samples. Interestingly, HSPA8 levels were significantly increased in OA cartilage relative to normal samples. HSPA8 was reported before to be downregulated in response to ER stress. To test if the profile of the key transcription factors ATF3 and CHOP/DDIT3, is affected in OA synovium similar to what we observed in cartilage, we analyzed three RNA sequencing studies (GDS2126, GDS5401, and GDS5403) comparing OA synovial biopsies with normal synovial biopsies. Interestingly, the expression levels of these key genes were markedly reduced in OA samples compared to normal counterparts consistent with the same observation in cartilage (supplemental Fig. S6*B*).

These data highlight a significant disruption of expression of genes necessary for ER homeostasis which may indicate that ER homeostasis is affected in osteoarthritis.

## Discussion

Injury to articular cartilage is a key risk factor in the initiation and progression of osteoarthritis, a degenerative disease with no modifying therapies identified to date (Reviewed in (43, 44)). Identifying the early cellular responses to injury would facilitate understanding the early events that drive the tissue damage and consequently the loss of function in this disease. Our data highlights a significant ubiquitin signature of articular cartilage modulated by mechanical injury. Proteins significantly enriched were key regulators in ubiquitin system and ERAD response. However, while the ubiquitin system is implicated in several diseases, to date, no studies have focused on its role in articular cartilage damage and in osteoarthritis. Here, we present for the first time the unique ubiquitin signature of cartilage injury and report the possible involvement of ER stress in modulating cellular responses that cause injury-induced tissue damage in articular cartilage.

In this study, we propose a model of early responses to tissue injury that causes enhanced ubiquitination events and involves key proteins that regulate ER stress and other cellular responses. The key players of ER stress VCP, Ubiquilin 1, and RAD23B together with the ER associated DUBs YOD1 and ATXN3 were differentially ubiquitinated following injury in our ubiquitomics experiments (Figs. 1, 4 and 5). These results were validated by ubiquitin enrichment pull-down assays followed by Western blotting. Interestingly, these ubiquitination events were very rapid and affected downstream proteins involved in ER stress response.

Through the ER stress adaptive response, the cell maintains physiological homeostasis and survival in response to stresses, including metabolic disturbance, reduced calcium levels, augmented oxidative stress, and the accumulation of unfolded or misfolded proteins. This response causes a translational attenuation accompanied by activation of the ERAD response and activation of chaperones gene expression. However, excessive and prolonged activation of the ER stress response causes cellular death (45, 46). Interrogating our previously published microarray data of murine hip cartilage, we observed that injury to hip cartilage causes a significant upregulation in the expression levels of ER stress–related mouse genes including Atf3, Atf4, Atf5, Ddit4, Trib3, Hspa1a &b, and Ddit3/CHOP (Fig. 7*A*). We also observed that injury to porcine cartilage induces gene expression levels of ER stress genes GRP78/BIP, XBP1, and CHOP. CHOP and GRP78 proteins were upregulated after injury in zebrafish embryos as well as at gene expression level in porcine cartilage following injury (Fig. 7). This indicates activation of the ATF6–CHOP–GRP78 axis in the ERAD response. We predict that this rapid response is to adapt to the cellular stress induced by injury and may induce repair. Interestingly, we also observed significant ubiquitination and enhanced gene expression levels of DDIT4, HSPa1B (supplemental Table S3 and data not shown) after injury highlighting the involvement of a wider range of ER stress–linked proteins to the injury cellular responses. However, we also observed that osteoarthritic tissues (cartilage or synovium) seem to fail to maintain this response and the ER stress genes are significantly reduced in diseased tissue compared to controls (supplemental Fig. S6). This highlights a possible disruption of ER homeostasis in OA, a mechanism that requires further investigation and potential druggable target development.

Our findings that injury rapidly caused a differential ubiquitination and activation of the ER-associated DUBs YOD1 and ATXN3 indicated that this change in ubiquitination pattern might directly regulate protein activity. Many DUBs are associated with E3 ligases that have an intrinsic tendency to self-ubiquitinate. This interaction allows stabilization of the E3 ligase by reversing its auto-ubiquitination and preventing its degradation. Likewise, DUBs are also targets for ubiquitination by E3 ligases (8, 9, 10, 47, 48, 49, 50, 51). E3–DUB interactions may allow fine-tuning of the ubiquitination status of a common substrate (7, 49). Ubiquitination of DUBs may regulate their catalytic activity by either competing with binding of ubiquitinated substrates especially when associated with an E3 ligase or by enhancing activity through activating conformational changes (8, 9, 10). The ubiquitination of ATXN3 at lysine 117 was reported to enhance its activity *in vitro* (8) and *in vivo* (52). The ubiquitination of YOD1 at lysine 213/257 is a novel observation that is not reported previously. We observed that YOD1 is mostly deubiquitinated after injury (Fig. 4*C*) and this may have caused the enhanced activity of a shifted form of the protein as observed in Figure 6*E*. This high molecular weight form could be a dimer or a multimer.

Interestingly, we observed that YOD1 and ATXN3 are also active in osteoarthritic cartilage (Fig. 6*H*), highlighting a possible sustained activation of these two DUBs following repetitive injury in diseased tissue. This implicates DUBs in modulation of inflammatory cellular responses to localized connective tissue injury. Further investigation of the role of these DUBs in modulating the pathological injury and disease progression *in vivo* is a crucial future prospective for validating these data *in vivo*. Another important question is if modulating DUB activity would interfere with cartilage degradation *in vivo* or *in vitro*. The role of these modified sites in cartilage development and function *in vivo* could be addressed in future mutational studies. DUBs are attractive therapeutic targets in several diseases, and we expect in the near future a number of clinical trials would include the use of DUB inhibitors or drugs that showed anti-DUB properties in osteoarthritis patients. The identification of specific inhibitors for injury-induced DUBs is another promising new avenue in disease targeting.

Overall, in this study, we identify a unique ubiquitin signature associated with injury cellular responses with key proteins involved in important cellular processes including endocytosis, carbon metabolism, biosynthesis of amino acids, and protein processing in ER. A specific set of proteins incorporating DUBs and ER stress regulators is also identified to be modulated by ubiquitination. We show that injury-dependent differential ubiquitination facilitated the activation of the three DUBs USP5, YOD1, and ATXN3. The early cellular response to injury involves ER stress markers activation. Using data mining approaches, we observed that ER stress genes are significantly downregulated in human osteoarthritic cartilage while negative regulators of ER stress as HSPA8 were significantly upregulated, highlighting a disrupted ER homeostasis in OA. The work presented here provides a number of significant clues of the early cellular responses to tissue injury and potential upstream regulators. Furthermore, data provides a platform for further investigation of these pathways and their role in tissue damage and repair. These pathways could be modulated to delay or inhibit tissue damage in diseases induced by pathological injury such as osteoarthritis.

## Data availability

The mass spectrometry proteomics and ubiquitomics data have been deposited to the ProteomeXchange Consortium via the PRIDE (53) partner repository with the data set identifier PXD030817. Annotated spectra of GlyGly(K)-modified peptides could be viewed through MS-Viewer Protein Prospector suite of software at http://prospector2.ucsf.edu/prospector/cgi-bin/msform.cgi?form=msviewer, with the search key: iadtjnlpr2. Any further data generated or analyzed during this study are included in this published article [and its supplementary information files]. The data sets analyzed for this study can be found in gene expression omnibus (GEO) under accession numbers GSE114007, GDS2126, GDS5401, GDS5403, and GSE155892.

## Supplemental data

This article contains supplemental data.

## Conflict of interest

The authors declare that they have no competing interests.
