# Supplementary material for: The Mechano-Ubiquitinome of Articular Cartilage: Differential Ubiquitination and Activation of a Group of ER-Associated DUBs and ER Stress Regulators
Source: Mol Cell Proteomics. 2022 Sep 28;21(12):100419. doi: 10.1016/j.mcpro.2022.100419 (PMC9708921; doi:10.1016/j.mcpro.2022.100419)
Supplement: Supplementary Figure S1 [file mmc1.pdf]

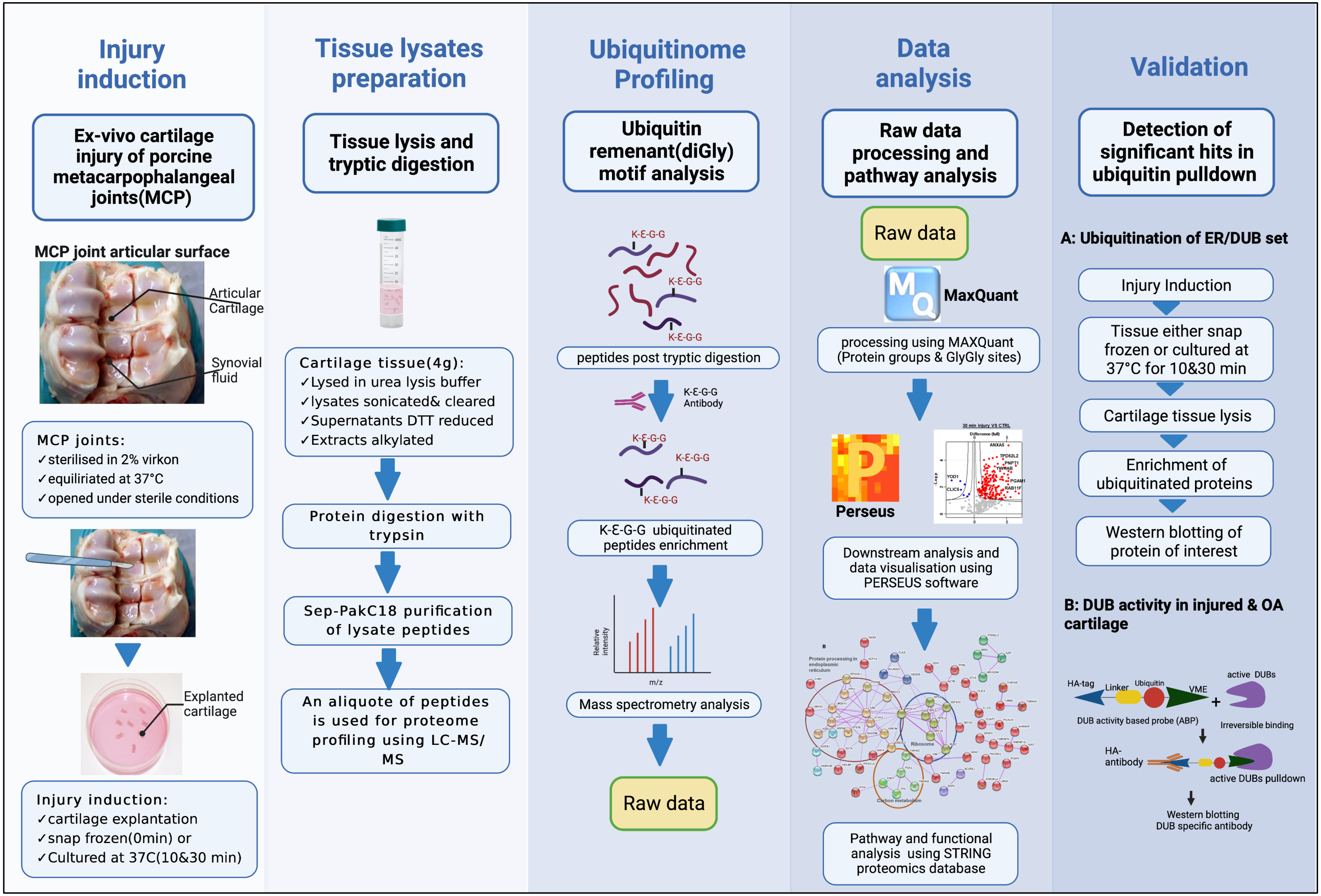

Supplementary Figure S1: Ex-vivo cartilage injury model and experimental workflow to detect ubiquitinome and proteome profiling of porcine articular cartilage post mechanical tissue injury
