# Supplementary material for: The Mechano-Ubiquitinome of Articular Cartilage: Differential Ubiquitination and Activation of a Group of ER-Associated DUBs and ER Stress Regulators
Source: Mol Cell Proteomics. 2022 Sep 28;21(12):100419. doi: 10.1016/j.mcpro.2022.100419 (PMC9708921; doi:10.1016/j.mcpro.2022.100419)
Supplement: Supplementary Figure S2 [file mmc2.pdf]

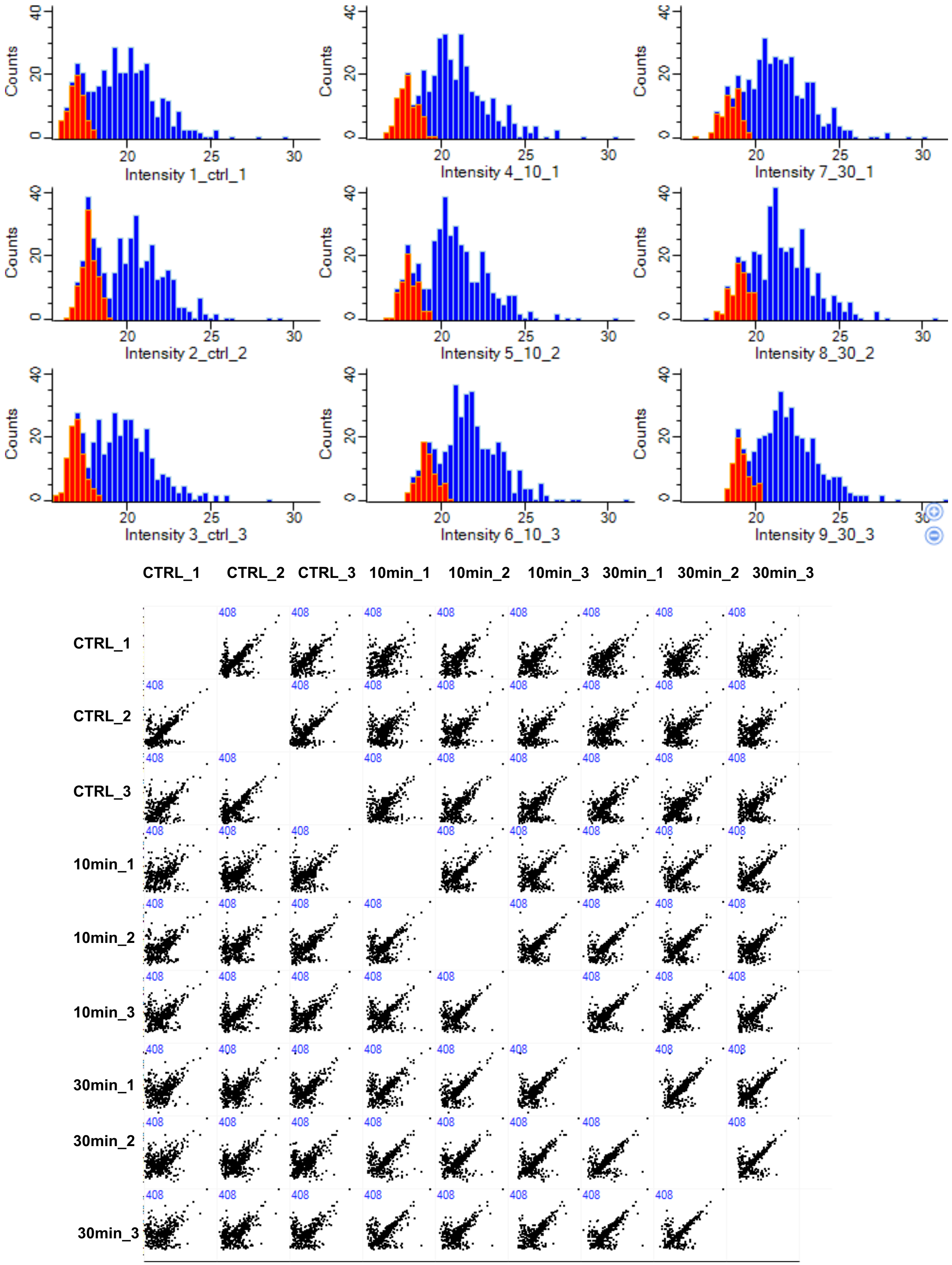

Supplementary Figure S2: Histogram analysis and scatter plot of individual cartilage samples in ubiquitinome analysis. Missing values from normal distribution were imputed as described in methods(red color). Numbers on Scatter plot shows peptides of valid values in all samples(408).
