# Supplementary material for: The Mechano-Ubiquitinome of Articular Cartilage: Differential Ubiquitination and Activation of a Group of ER-Associated DUBs and ER Stress Regulators
Source: Mol Cell Proteomics. 2022 Sep 28;21(12):100419. doi: 10.1016/j.mcpro.2022.100419 (PMC9708921; doi:10.1016/j.mcpro.2022.100419)
Supplement: Supplementary Figure S3 [file mmc3.pdf]

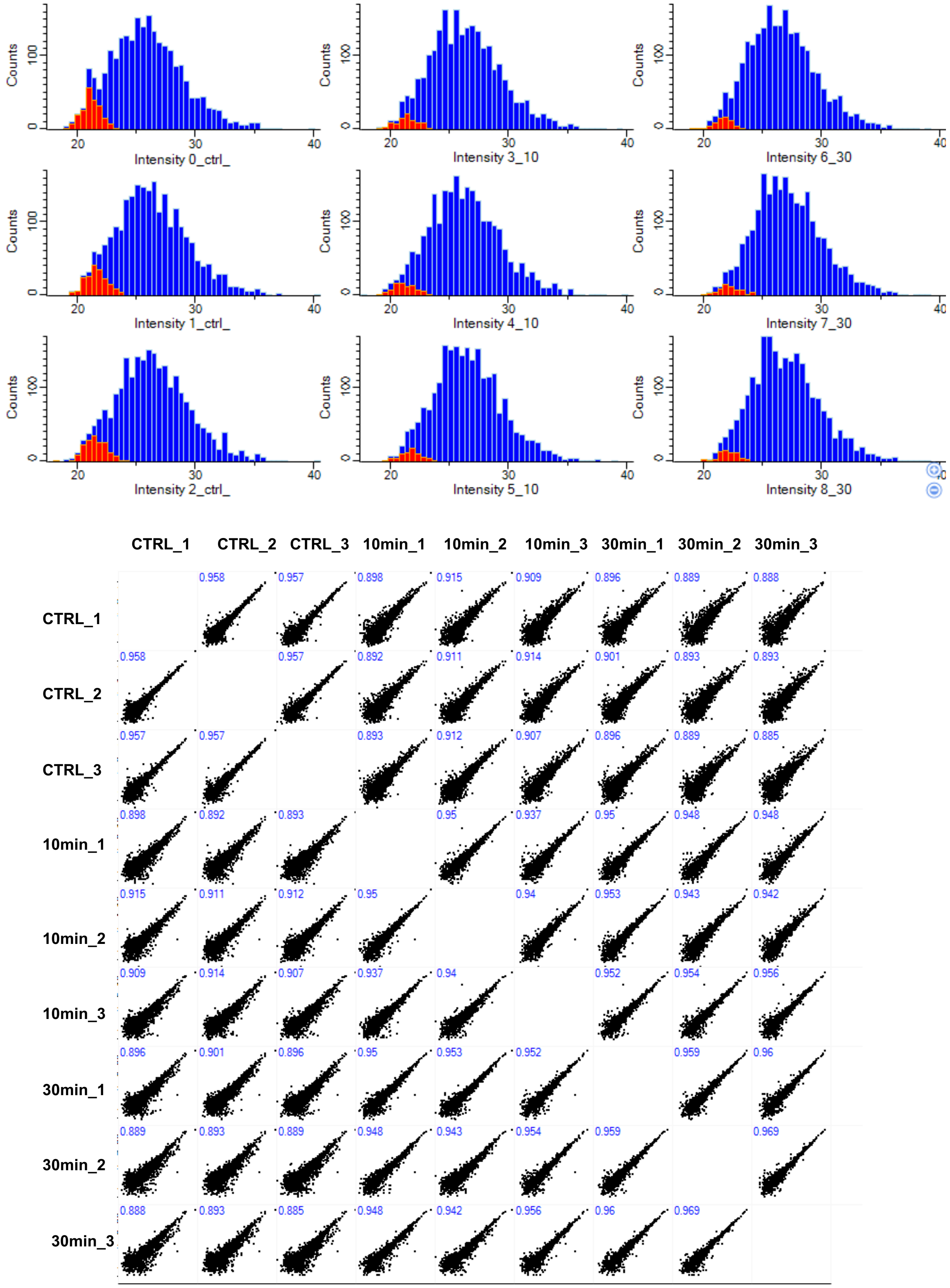

Supplementary Figure S3: Histogram analysis and scatter plot of individual samples in proteome dataset. Missing values from normal distribution were imputed as described in methods (red color). Numbers on Scatter plot shows Pearson correlation between individual samples.
