# Supplementary material for: The Mechano-Ubiquitinome of Articular Cartilage: Differential Ubiquitination and Activation of a Group of ER-Associated DUBs and ER Stress Regulators
Source: Mol Cell Proteomics. 2022 Sep 28;21(12):100419. doi: 10.1016/j.mcpro.2022.100419 (PMC9708921; doi:10.1016/j.mcpro.2022.100419)
Supplement: Supplementary Figure S4 [file mmc4.pdf]

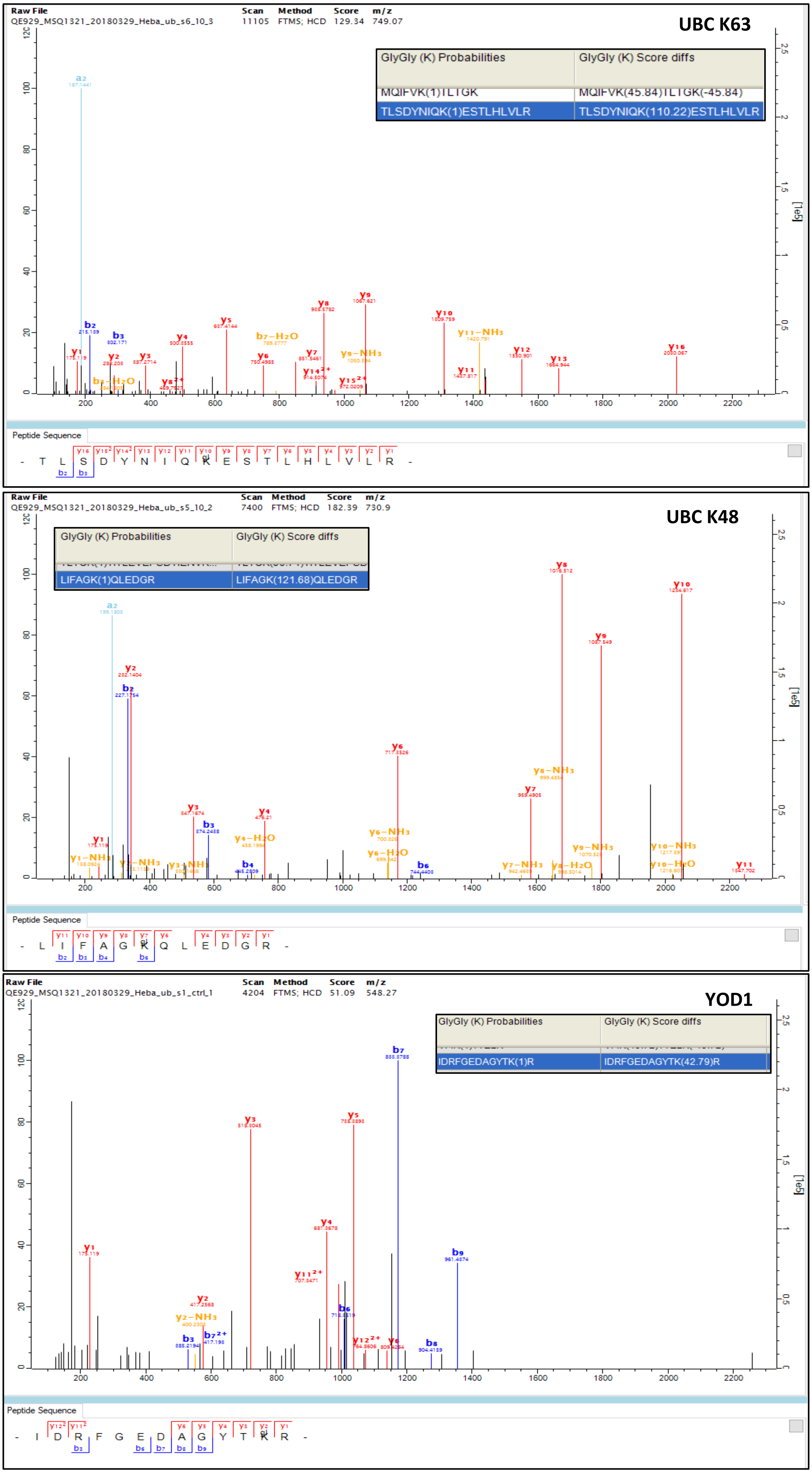

Supplementary Figure S4: Annotated spectra of enriched ubiquitinated peptides lysine 63 and lysine 48 of ubiquitin protein(UBC) and YOD1. Spectra are visualised using Viewer in Maxquant software package version 1.6.7.0.
