# Supplementary material for: The Mechano-Ubiquitinome of Articular Cartilage: Differential Ubiquitination and Activation of a Group of ER-Associated DUBs and ER Stress Regulators
Source: Mol Cell Proteomics. 2022 Sep 28;21(12):100419. doi: 10.1016/j.mcpro.2022.100419 (PMC9708921; doi:10.1016/j.mcpro.2022.100419)
Supplement: Supplementary Figure S5 [file mmc5.pdf]

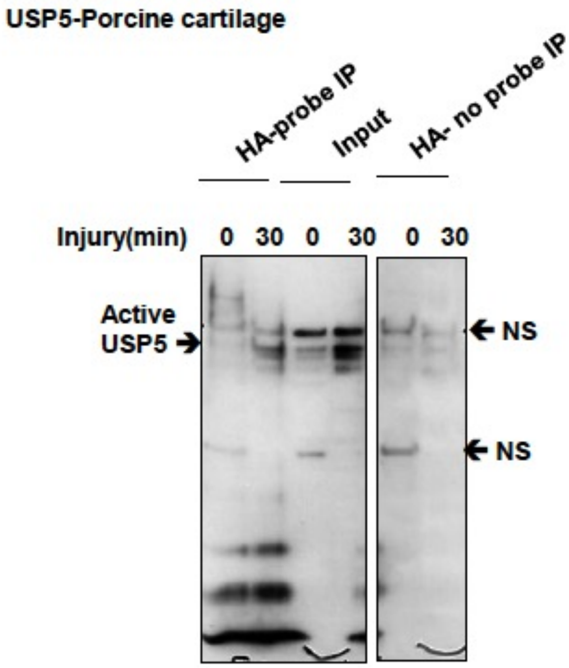

Supplementary Figure S5: USP5 deubiquitinase is activated in response to articular cartilage injury. Porcine cartilage was lysed as described in methods. 50 µg of lysates were reacted with HA DUB activity probe and for 45 minutes at 37°C and then subjected to immunoprecipitation of active DUBs using anti HA antibody conjugated magnetic beads. Immunocomplexes were analysed by western blotting and compared with input and IP control using USP5 DUB specific antibody. NS: non specific
