# Supplementary material for: The Mechano-Ubiquitinome of Articular Cartilage: Differential Ubiquitination and Activation of a Group of ER-Associated DUBs and ER Stress Regulators
Source: Mol Cell Proteomics. 2022 Sep 28;21(12):100419. doi: 10.1016/j.mcpro.2022.100419 (PMC9708921; doi:10.1016/j.mcpro.2022.100419)
Supplement: Supplementary Figure S6 [file mmc6.pdf]

A: OA cartilage: GSE114007

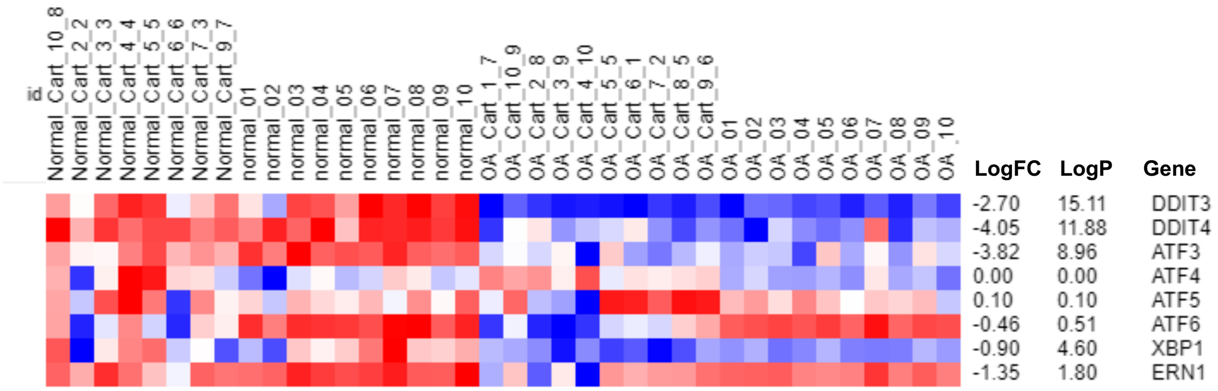

B: OA Synovium

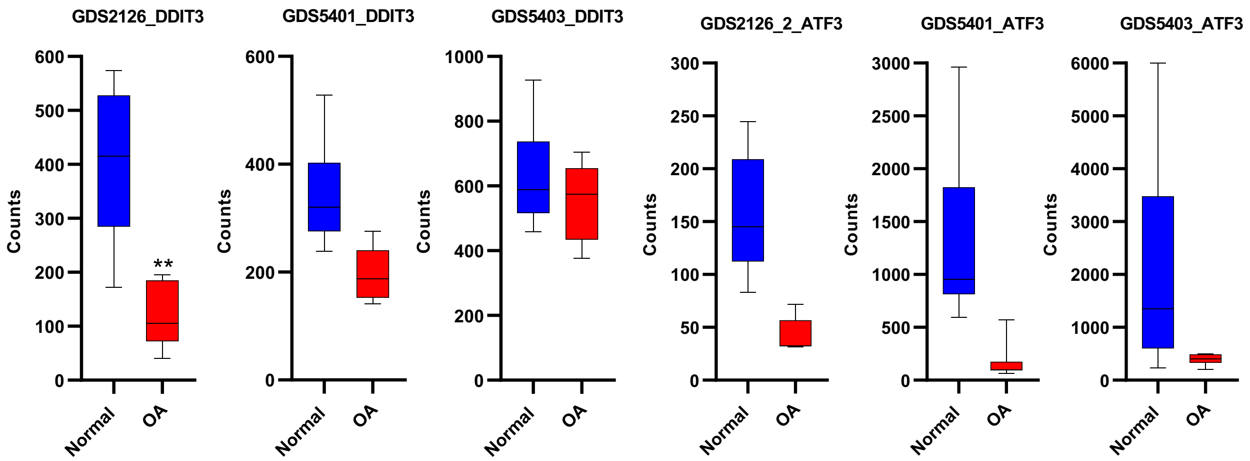

Supplementary Figure S6: Modulation of ER stress responsive genes in osteoarthritic cartilage and synovium tissues compared to normal tissues. RNA counts were retrieved from Gene Expression Omnibus studies as following **GSE114007 RNA seq study in OA cartilage** versus normal donors (38 donors : 18 normal and 20 OA)(PMID: 30081074), GDS5403: 33 samples: 10 normal, 10 OA , 13 RA(PMID: 24690414) and GDS5401: Berlin data set (10 normal, 10 OA, and 10 RA)(PMID: 24690414)
