# Supplementary material for: The Mechano-Ubiquitinome of Articular Cartilage: Differential Ubiquitination and Activation of a Group of ER-Associated DUBs and ER Stress Regulators
Source: Mol Cell Proteomics. 2022 Sep 28;21(12):100419. doi: 10.1016/j.mcpro.2022.100419 (PMC9708921; doi:10.1016/j.mcpro.2022.100419)
Supplement: Supplementary Table S2 [file mmc8.pdf]

No	Oligo Name	Sequence (5'->3')
1	PIG-CHOP_F	CTTTCTCCTTCGGGACACTG
2	PIG-CHOP_R	GGGAGGTGTGTGTGACCTCT
3	PIG-XBP1-F	TAGCAGCTCAGACTGCCAGA
4	PIG-XBP1-R	GTCTGGGGAAGGACATCTGA
5	PIG-GAPDH-F	CCCTGTCACCCCTTAACAGA
6	PIG-GAPDH-R	GTTAAAAGCAGCCCTGGTGA
7	PIG-GAPDH-F2	CATGGAGAAGGCTGGGGCTC
8	PIG-GAPDH-R2	ATGAGGTCCACCACCCTGTT
9	PIG-BIP-F	TGGGTTGAATGTCATGAGGA
10	PIG-BIP-R	CGCTGGTCAAAGTCTTCTCC
